# Resveratrol ameliorates prenatal progestin exposure-induced autism-like behavior through ERβ activation

**DOI:** 10.1186/s13229-018-0225-5

**Published:** 2018-08-02

**Authors:** Weiguo Xie, Xiaohu Ge, Ling Li, Athena Yao, Xiaoyan Wang, Min Li, Xiang Gong, Zhigang Chu, Zhe Lu, Xiaodong Huang, Yun Jiao, Yifei Wang, Meifang Xiao, Haijia Chen, Wei Xiang, Paul Yao

**Affiliations:** 1 Institute of Rehabilitation Center, Tongren Hospital of Wuhan University, Wuhan, 430060 People’s Republic of China; 2 SALIAI Stem Cell Institute of Guangdong, Guangzhou SALIAI Stem Cell Science and Technology Co. LTD, Guangzhou, 510055 People’s Republic of China; 3 Department of Pediatrics, Hainan Maternal and Child Health Hospital, Haikou, 570206 People’s Republic of China

**Keywords:** Autism spectrum disorder, Estrogen receptor β, Lipid metabolism, Mitochondria, Oxidative stress, Progestin, Resveratrol

## Abstract

**Background:**

Recent literatures indicate that maternal hormone exposure is a risk factor for autism spectrum disorder (ASD). We hypothesize that prenatal progestin exposure may counteract the neuroprotective effect of estrogen and contribute to ASD development, and we aim to develop a method to ameliorate prenatal progestin exposure-induced autism-like behavior.

**Methods:**

Experiment 1: Prenatal progestin exposure-induced offspring are treated with resveratrol (RSV) through either prenatal or postnatal exposure and then used for autism-like behavior testing and other biomedical analyses. Experiment 2: Prenatal norethindrone (NET) exposure-induced offspring are treated with ERβ knockdown lentivirus together with RSV for further testing. Experiment 3: Pregnant dams are treated with prenatal NET exposure together with RSV, and the offspring are used for further testing.

**Results:**

Eight kinds of clinically relevant progestins were used for prenatal exposure in pregnant dams, and the offspring showed decreased ERβ expression in the amygdala with autism-like behavior. Oral administration of either postnatal or prenatal RSV treatment significantly reversed this effect with ERβ activation and ameliorated autism-like behavior. Further investigation showed that RSV activates ERβ and its target genes by demethylation of DNA and histone on the ERβ promoter, and then minimizes progestin-induced oxidative stress as well as the dysfunction of mitochondria and lipid metabolism in the brain, subsequently ameliorating autism-like behavior.

**Conclusions:**

We conclude that resveratrol ameliorates prenatal progestin exposure-induced autism-like behavior through ERβ activation. Our data suggest that prenatal progestin exposure is a strong risk factor for autism-like behavior. Many potential clinical progestin applications, including oral contraceptive pills, preterm birth drugs, and progestin-contaminated drinking water or seafood, may be risk factors for ASD. In addition, RSV may be a good candidate for clinically rescuing or preventing ASD symptoms in humans, while high doses of resveratrol used in the animals may be a potential limitation for human application.

**Electronic supplementary material:**

The online version of this article (10.1186/s13229-018-0225-5) contains supplementary material, which is available to authorized users.

## Background

Autism spectrum disorder (ASD) is a neurological and developmental disorder that is characterized by deficits in social communication and interaction with restricted and repetitive patterns of behavior [1]. The prevalence of ASD is estimated to be 1:68 and biased towards males with a male-to-female ratio of at least 4:1 [2, 3]. Many risk factors contribute to ASD development, including genetics, sex, and environmental factors [4, 5], while the detailed mechanisms of ASD remain unclear [6].

Recent literatures have shown that dysregulation of estrogen receptor β (ERβ) is associated with ASD [7–10]. ERβ regulates the basal expression of superoxide dismutase (SOD2), which regulates oxidative stress [11], and estrogen-related receptor α (ERRα) [12], which regulates mitochondrial function and lipid metabolism [13, 14]. ERβ suppression results in oxidative stress and dysfunction of mitochondrial and lipid metabolism, subsequently triggering brain damage and autism-like behavior [9, 15]. This indicates that hormone-mediated ERβ suppression may contribute to ASD development.

It has been reported that maternal hormonal exposure is a significant risk factor for ASD [8, 16] as steroidogenic activity is elevated in some ASD patients [17] and cholesterol metabolism and various steroid abnormalities are involved in ASD development [18]. In addition, natural progesterone and synthetic progestin regulate neurogenic responses [19] and impair cognitive flexibility during development [20] as well as downregulate ERβ expression [9, 21]. We hypothesize that clinically relevant progestin may counteract estrogen-mediated neuroprotective effects via downregulation of ERβ, and contribute to ASD development [22, 23].

Resveratrol (RSV) is a natural polyphenolic compound that is present at high levels in red grapes, nuts, pomegranates, and *Polygonum cuspidatum* [24]. Resveratrol (RSV) has much therapeutic potential with its antioxidant, antitumorigenic, and cardioprotective as well as neuroprotective effects [24–28]. All of these biological activities may have potential benefits and points of interest in autism therapeutics, although very little research has been reported on its potential effect on ASD treatment [15, 29, 30].

In this study, different kinds of clinically relevant progestins were used for prenatal exposure in pregnant dams, and the offspring showed decreased ERβ expression in the brain with autism-like behavior. Oral administration of resveratrol (RSV) by either postnatal or prenatal treatment completely reversed this effect with ERβ activation and ameliorated autism-like behavior. Further investigation showed that RSV-mediated ERβ activation is due to RSV-mediated demethylation of DNA and histone on the ERβ promoter. This is the first time we have discovered the potential mechanism of ASD development due to prenatal progestin exposure-induced ERβ suppression, as well as the potential rescuing and preventive effect of RSV on autism-like behavior through ERβ activation, which may potentially be applicable in clinical treatment of ASD patients.

## Methods

A detailed description can be found in Additional file 1.

### Materials

17β-estradiol (E2, #E2758); progesterone (P4, #P0130); levonorgestrel (LNG, #1362602); medroxyprogesterone acetate (MPA, #1378001); nestorone (NES, # SML0550); norethindrone (NET, #1469005); norethindrone acetate (NETA, #1470004); norgestimate (NGM, # 1471914); hydroxyprogesterone caproate (OHPC, #1329006), and resveratrol (RSV, #R5010) were obtained from Sigma. Norethynodrel (NEN, #E4600–000) was obtained from Steraloids.

### In vivo rat experiments

The animal protocol conformed to the US NIH guidelines (Guide for the Care and Use of Laboratory Animals, No. 85–23, revised 1996) and was reviewed and approved by the Institutional Animal Care and Use Committee from Wuhan University [9].

#### Protocol 1 for postnatal treatment of resveratrol

**Fig. 1:**
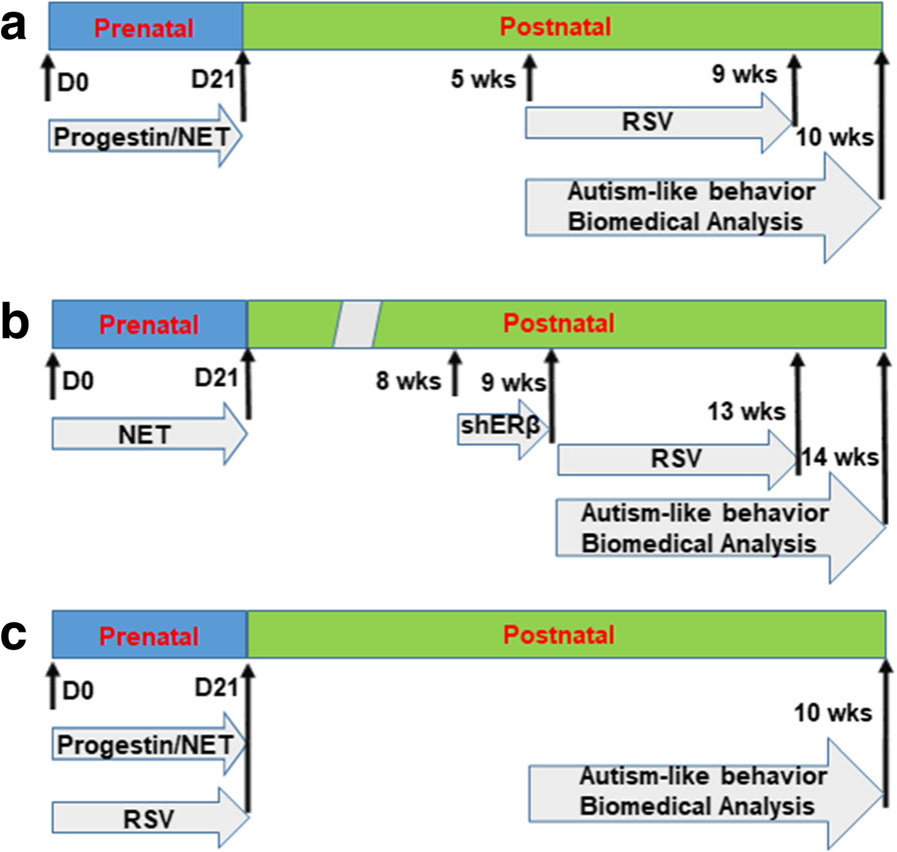
Schematic details for experimental design in this study. **a** Protocol 1 for postnatal treatment of resveratrol. **b** Protocol 2 for postnatal treatment of resveratrol with infusion of shERβ lentivirus. **c** Protocol 3 for prenatal treatment of resveratrol

Three-month-old female Sprague Dawley rats were caged with proven males, and the verified pregnant dams were randomly assigned to either 20 mg of progestin (such as norethindrone, NET) or VEH (vehicle group that received the same volume of vehicle). Drugs were suspended in 5% ethanol in organic sesame oil and 0.1 ml was given daily through subcutaneous injection at the nape starting from day 1 until pup delivery for ~ 21 days. The male and female offspring were separated from the dams on day 21, and then at 5 weeks old, the offspring from either VEH or progestin prenatal treatment were randomly divided into two groups, a resveratrol (RSV) group and a control (CTL) group. Rats in the RSV group were orally administered (by gavage) 20 mg/kg of RVS suspended in 10 g/l carboxymethylcellulose every day for 4 weeks (28 days). Those in the CTL group were administered 10 ml/kg of 10 g/l carboxymethylcellulose during the same period. At 10 weeks old, treated offspring were used for autism-like behavior testing or were sacrificed for further experiments and biomedical analysis [9]; see schematic details in Fig. 1a.

#### Protocol 2 for postnatal treatment of resveratrol with infusion of shERβ lentivirus

The male offspring (8 weeks old) from the VEH and NET group in Protocol 1 were anesthetized with a mixture of ketamine (90 mg/kg) and xylazine (2.7 mg/kg) and implanted with a guide cannula targeting the amygdala (26 gauge; Plastics One). The following coordinates were chosen for the amygdala: − 2.0 mm posterior to the bregma, ± 4.2 mm from the midline, and − 7.2 mm from the skull surface on which it was based. Cannula was attached to the skull with dental acrylic and jeweler’s screws and closed with an obturator [31]. An osmotic minipump (Alzet model 2002; flow rate 0.5 μl/h; Cupertino, CA) connected to a 26-gauge internal cannula that extended 1 mm below the guide was implanted and used to deliver either ERβ knockdown (shERβ), or empty (EMP) lentivirus. Vehicle consisting of artificial cerebrospinal fluid (aCSF; 140 mM NaCl, 3 mM KCl, 1.2 mM Na_2_HPO_4_, 1 mM MgCl_2_, 0.27 mM NaH_2_PO_4_, 1.2 mM CaCl_2_, and 7.2 mM dextrose, pH 7.4) was used for the infusion of the lentivirus. Infusion (flow rate 0.5 μl/h) began immediately after placement of the minipump. 0.5 μl of total 2 × 10^3^ cfu of lentivirus was infused for 1 h [9]. The experimental rats were separated into four groups (12 per group) at 9 weeks old. Group 1: VEH offspring with empty control lentivirus infusion plus oral administration of carboxymethylcellulose control treatment for 4 weeks (VEH/EMP/CTL); Group 2: NET offspring with empty control lentivirus infusion plus oral administration of carboxymethylcellulose control treatment for 4 weeks (NET/EMP/CTL); Group 3: NET offspring with empty control lentivirus infusion plus oral administration of resveratrol (RSV) treatment for 4 weeks (NET/EMP/RSV); Group 4: NET offspring with ERβ knockdown lentivirus infusion plus oral administration (by gavage) of RSV treatment for 4 weeks (VEH/shERβ/RSV). At 14 weeks old, the offspring were used for behavior testing followed by biomedical analysis [9]; see schematic details in Fig. 1b.

#### Protocol 3 for prenatal treatment of resveratrol

Three-month-old verified pregnant dams were randomly assigned to the following four groups: Group 1: The rats received VEH (5% ethanol in organic sesame oil and 0.1 ml were given daily through subcutaneous injection at the nape) plus control (CTL) group with oral administration of 10 ml/kg of 10 g/l carboxymethylcellulose (VEH/PreCTL); Group 2: Rats received 20 μg of progestin (such as norethindrone, NET) with 5% ethanol in organic sesame oil plus) plus CTL group (NET/PreCTL); Group 3: The rats received VEH treatment plus resveratrol (RSV) group with oral administration (by gavage) of 20 mg/kg of resveratrol suspended in 10 g/l carboxymethylcellulose (VEH/PreRSV); Group 4: The rats received progestin (such as NET) group plus RSV group (NET/PreRSV). The rats received the above treatment starting from day 1 until pup delivery for ~ 21 days. Both male and female offspring were raised until 10 weeks old to be tested for autism-like behavior testing and other biomedical analysis [9], see schematic details in Fig. 1c.

### Animal behavior test

The animal behavior test of offspring was carried out at 10 weeks of age. Female offspring were tested in the diestrus phase, which was confirmed by vaginal smears. Autism-like behavior was evaluated using the marble burying test (MBT) and social interaction (SI) test.

#### Marble burying test (MBT)

In brief, each rat is placed in a clean cage (35 × 23 × 19 cm^3^) filled with wood chip bedding to a depth of 5 cm containing 20 colored glass marbles (1 cm diameter) placed in a 5 × 4 arrangement. The number of marbles buried (> 50% covered by bedding material) in 30 min was hand-scored by the experimenter [9, 32, 33].

#### Social interaction (SI) test

In short, the subjects (Test and Stranger) were separately habituated to the arena for 5 min before the test. During each test, the rats were placed into the apparatus over a period of 20 min and the time spent following, mounting, grooming, and sniffing any body parts of the other rat was taken as an indicator of social engagement, and the social interaction time was calculated and analyzed using EthoVision XT animal tracking software (Noldus, USA) [34]. The animal used as the “Stranger” was used only once and was a Sprague Dawley rat of the same gender, weight, and age, with no previous contact with the test rats [9, 32, 33].

### Methods

The amygdala neurons were isolated for in vitro primary cell culture analysis [9, 35]. The DNA methylation on the rat ERβ promoter was evaluated by a real-time PCR-based methylation-specific PCR (MSP) analysis as described previously with some modifications [9, 36–38]. The lentivirus for rat ERβ shRNA and empty control (EMP) were prepared previously in our lab [9]. The mRNA was measured by real-time quantitative PCR using primers provided in Additional file 1: Table S1, and the protein was measured by western blotting [9]. The SIRT1 activity assay was evaluated in nuclear extract using a SIRT1 Fluorometric Drug Discovery Kit (Cat #: BML-AK555, Enzo Life Sciences) [39]. Oxidative stress was evaluated by in vivo superoxide anion (O_2_^−^) release [40] and 3-nitrotyrosine formation. DNA damage was evaluated by 8-OHdG and γH2AX formation [9]. Mitochondrial function was evaluated by mitochondrial DNA copies [9] and intracellular ATP level [40]. The histone methylation was evaluated by chromatin immunoprecipitation (ChIP) analysis [9]. Fatty acid metabolism was evaluated by in vitro lipid transport assay [41] and fatty acid oxidation assay [42, 43]. Statistical analysis was conducted using SPSS 22 software, and a *P* value of < 0.05 was considered significant, and the data was given as mean ± SEM [9].

## Results

### Postnatal resveratrol treatment reverses prenatal progestin exposure-induced ERβ suppression and autism-like behavior

We first investigated the potential effect of prenatal progestin exposure on ERβ expression in the amygdala and autism-like behavior in 10-week-old male offspring, and then evaluated whether postnatal resveratrol treatment could reverse this effect. In Table 1 (left panel of first row), several steroids, 17β-estradiol (E2), progesterone (P4), and eight different kinds of clinically relevant progestins, including levonorgestrel (LNG), medroxyprogesterone acetate (MPA), nestorone (NES), norethindrone (NET), norethindrone acetate (NETA), norethynodrel (NEN), norgestimate (NGM), and hydroxyprogesterone caproate (OHPC), were used for prenatal exposure to different 3-month-old pregnant dams for 21 days (*n* = 8). The 5-week-old male offspring were then treated by either control (CTL) or resveratrol (RSV) for 4 weeks, and the ERβ mRNA in the amygdala was evaluated by real-time PCR and the autism-like behavior was evaluated by social interaction time. Our results showed that E2 had no effect, while P4 slightly decreased ERβ expression but had no significant effect on social interaction time, and almost all the clinically relevant progestins significantly decreased ERβ expression (except the NGM), and decreased social interaction time (except the NEN) compared to the vehicle group. Furthermore, resveratrol treatment almost completely reversed the suppression effect on ERβ expression and social interaction time (partly for the NES and NGM). Our results indicate that postnatal resveratrol treatment reverses prenatal progestin exposure-induced ERβ suppression and autism-like behavior.

**Table 1:** Resveratrol reverses and prevents prenatal progestin exposure-induced ERβ suppression and autism-like behavior

Rats	Postnatal treatment of RSV				Prenatal treatment of RSV			
Prenatal progestin exposure	ERβ mRNA level (%)		Social interaction time (seconds)		ERβ mRNA level (%)		Social interaction time (seconds)	
	Control	RSV	Control	RSV	Control	RSV	Control	RSV
Vehicle	100 ± 9	111 ± 10	351 ± 27	369 ± 17	100 ± 11	91 ± 11	356 ± 24	340 ± 16
E2	94 ± 12	91 ± 13	369 ± 24	343 ± 16	89 ± 13	95 ± 9	341 ± 17	336 ± 19
P4	78 ± 10*	119 ± 9	321 ± 17	339 ± 22	78 ± 8*	106 ± 8	319 ± 22	369 ± 23
LNG	54 ± 11*	89 ± 12	259 ± 19*	321 ± 19	61 ± 9*	109 ± 11	263 ± 12*	335 ± 17
MPA	72 ± 13*	115 ± 7	261 ± 24*	351 ± 28	66 ± 12*	92 ± 8	245 ± 11*	361 ± 22
NES	81 ± 11	91 ± 9	249 ± 20*	298 ± 23*	75 ± 8*	111 ± 12	266 ± 18*	350 ± 18
NET	49 ± 8*	106 ± 11	237 ± 16*	326 ± 20	55 ± 10*	106 ± 9	220 ± 25*	348 ± 20
NETA	67 ± 9*	90 ± 9	286 ± 19*	378 ± 14	72 ± 13*	113 ± 11	301 ± 17*	356 ± 25
NEN	75 ± 8*	113 ± 12	336 ± 26	361 ± 15	69 ± 14*	78 ± 9*	321 ± 25	336 ± 21
NGM	85 ± 12	90 ± 8	279 ± 22*	306 ± 22*	78 ± 9*	135 ± 11*	291 ± 23*	343 ± 24
OHPC	59 ± 10	109 ± 11	268 ± 19*	322 ± 21	63 ± 11*	106 ± 12*	287 ± 19*	326 ± 22

*n* = 8, results are expressed as mean ± SEM

Note: E2, 17β-estradiol; P4, progesterone; LNG, levonorgestrel; MPA, medroxyprogesterone acetate; NES, nestorone; NET, norethindrone; NETA, norethindrone acetate; NEN, norethynodrel; NGM, norgestimate; OHPC, hydroxyprogesterone caproate; RSV, resveratrol.

*P < 0.05, vs vehicle group in the same column

### Prenatal resveratrol treatment prevents prenatal progestin exposure-induced ERβ suppression and autism-like behavior

We then evaluated the potential effect of prenatal resveratrol treatment on prenatal progestin exposure-induced ERβ suppression and autism-like behavior. In Table 1 (right panel of first row), prenatal exposure of P4 and all the eight different kinds of clinically relevant progestins significantly decreased ERβ expression and social interaction time (except the NEN), while prenatal resveratrol treatment (oral administration of 20 mg/kg RSV for 21 days) almost completely reversed prenatal progestin exposure-induced suppression effect on ERβ expression (partly for NEN) and social interaction time. Our results indicate that prenatal resveratrol treatment prevents prenatal progestin exposure-induced ERβ suppression and autism-like behavior.

### Postnatal resveratrol treatment reverses prenatal norethindrone exposure-induced suppression of ERβ and its target genes in the amygdala

**Fig. 2:**
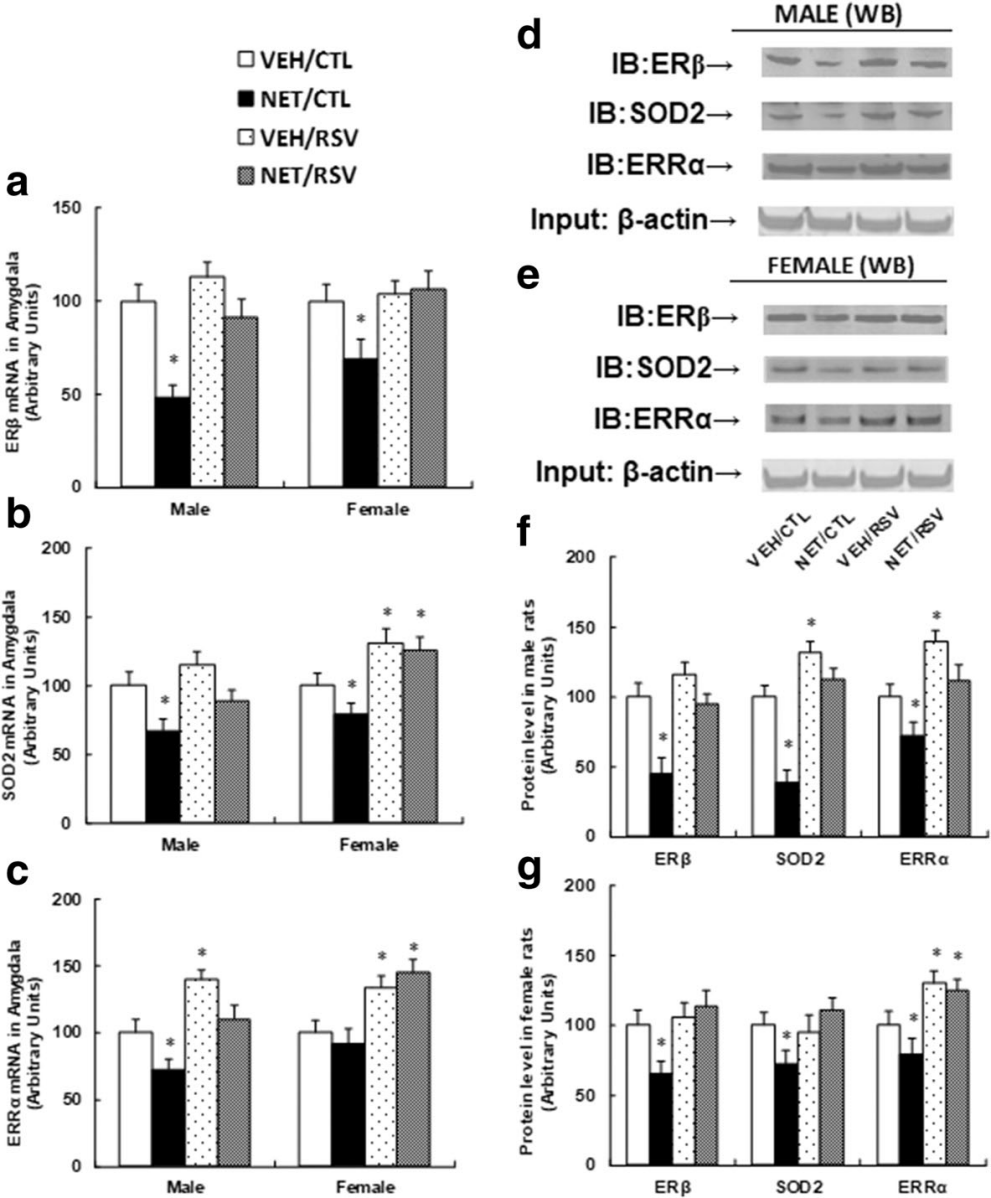
Postnatal resveratrol treatment reverses prenatal norethindrone exposure-induced suppression of ERβ and its target genes. Three-month-old pregnant dams were exposed to NET (20 μg norethindrone) or VEH (vehicle, 5% ethanol in organic sesame oil) by subcutaneous daily injection of 0.1 ml for 21 days until pup delivery. Both male and female offspring were then treated with either control (CTL) or resveratrol (RSV) for 4 weeks starting from 5 weeks old. The offspring were sacrificed at 10 weeks of age to isolate the amygdala for further analysis. **a–c** mRNA levels in the amygdala for genes of ERβ **a**, SOD2 **b**, and ERRα **c**, *n* = 5. **d** Representative pictures of protein levels in male offspring by western blotting. **e** Representative pictures of protein levels in female offspring by western blotting. **f** Protein expression quantitation for **d**, *n* = 5. **g** Protein expression quantitation for **e**, *n* = 5. *, *P* < 0.05, vs VEH/CTL group; ¶, *P* < 0.05, vs NET/CTL group. Results are expressed as mean ± SEM

In Table 1, the prenatal exposure of norethindrone (NET) in pregnant dams showed the most dramatic suppression effect on ERβ expression and social interaction time in offspring compared to other progestins. The NET was chosen for the prenatal progestin exposure in the later experiments to investigate the detailed mechanism and effect of resveratrol on autism-like behavior. The animal was treated as shown in Fig. 1b. We first measured the gene expression of ERβ, SOD2, and ERRα in the hypothalamus and hippocampus (see Additional file 1: Figure S1). We found that prenatal NET exposure showed no effect on gene expression. Also, RSV treatment showed no effect on the expression of ERβ and SIRT1, while the expression of SOD2 and ERRα was significantly increased in both the hypothalamus and hippocampus by RSV treatment compared to the control (CTL) group. We then measured the mRNA expression of ERβ (see Fig. 2a), SOD2 (see Fig. 2b), and ERRα (see Fig. 2c) in the amygdala. We found that RSV treatment completely reversed prenatal NET exposure-induced suppression of ERβ, SOD2, and ERRα in both male and female offspring. Furthermore, in female offspring, RSV significantly increased expression of SOD2 and ERRα in both the VEH and NET groups, while in male offspring there was much less gene activation (see Fig. 2b, c), indicating that female offspring seem more responsive to RSV treatment compared to male offspring. We also measured the protein levels in the amygdala (see Fig. 2d–g). The results showed that RSV completely reversed the NET-mediated suppression effect on the expression of ERβ, SOD2, and ERRα. Furthermore, RSV increased ERRα expression in VEH treatment compared to the CTL group in male offspring (see Fig. 2d, f), and in female offspring, RSV significantly increased ERRα expression in both VEH and NET treatment compared to CTL group (see Fig. 2e, g). We also measured the SIRT1 mRNA expression (see Figure S2a) and SIRT1 activity (see Figure S2b), which showed no difference in any of the treatments, indicating that SIRT1 may not be involved in RSV-mediated effect (see detailed statistical information in Additional file 1: Data S1). Our results suggest that RSV reverses prenatal NET exposure-induced suppression of ERβ and its target genes, and female offspring seem more responsive than male offspring.

### Postnatal resveratrol treatment diminishes prenatal norethindrone exposure-induced methylation of DNA and histone on the ERβ promoter

**Fig. 3:**
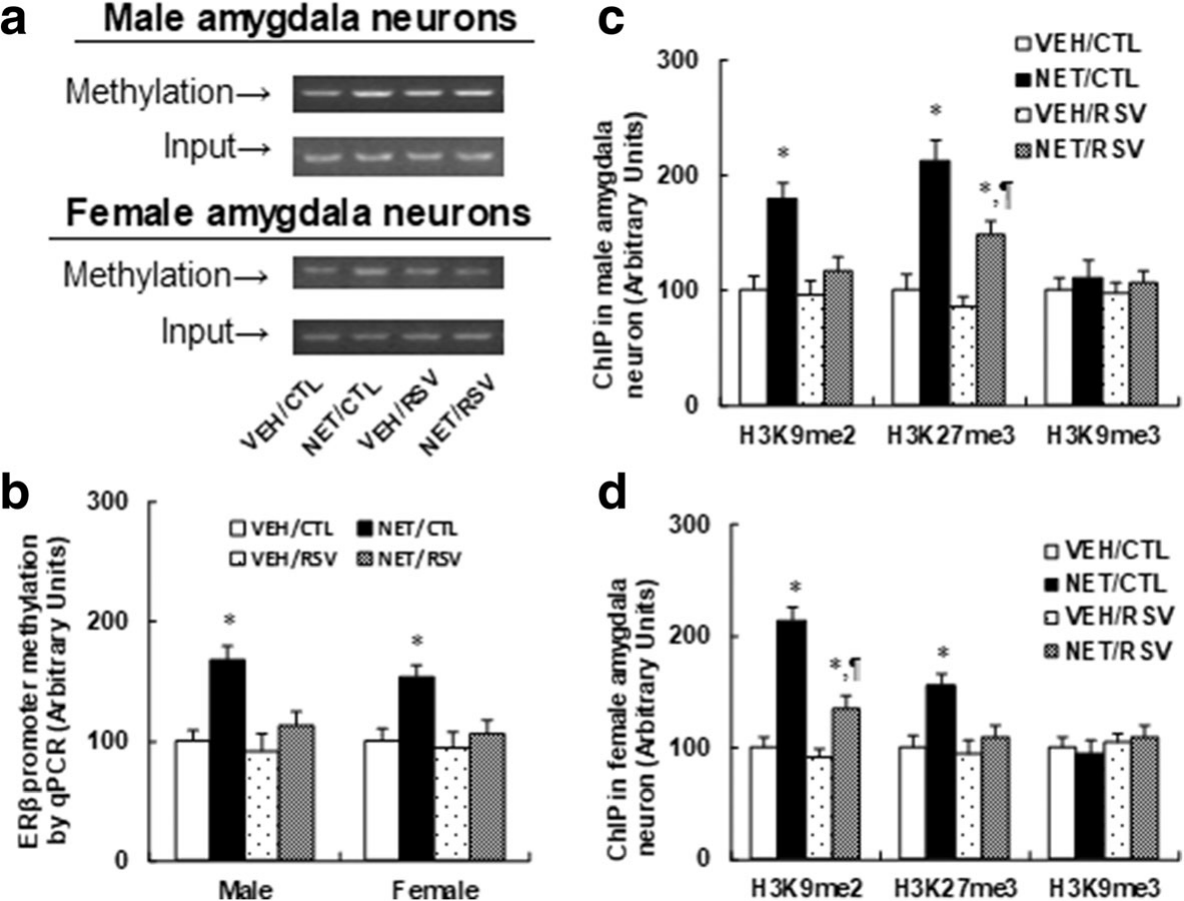
Postnatal resveratrol treatment diminishes prenatal norethindrone exposure-induced methylation of DNA and histone on the ERβ promoter. The amygdala neurons were isolated from 10-week-old male/female offspring for in vitro cell culture analysis. **a** The representative bands for ERβ methylation in amygdala neurons from both male (upper panel) and female (lower panel) offspring. **b** DNA methylation on ERβ by real-time PCR-based methylation-specific PCR (MSP) analysis in amygdala neurons, *n* = 5. **c** ChIP analysis on the ERβ promoter in male amygdala neurons, *n* = 5. **d** ChIP analysis on the ERβ promoter in female amygdala neurons, *n* = 5. *, *P* < 0.05, vs VEH/CTL group; ¶, *P* < 0.05, vs NET/CTL group. Results are expressed as mean ± SEM

In Fig. 3a, b, the DNA methylation on the ERβ promoter was significantly increased by prenatal NET exposure, and RSV treatment completely diminished this effect compared to the CTL group. We then measured the histone methylation on the ERβ promoter by ChIP techniques. In male offspring (see Fig. 3c), prenatal NET exposure increased H3K9 di-methylation (H3K9me2) by 1.80-fold and H3K27 tri-methylation (H3K27me3) by 2.13-fold, while in female offspring (see Fig. 3d), it increased H3K9 di-methylation (H3K9me2) by 2.14-fold and H3K27 tri-methylation (H3K27me3) by 1.56-fold, but had no effect on H3K9 tri-methylation (H3K9me3). RSV treatment completely diminished the effect on H3K9me2, but partly diminished the effect on H3K27me3 in male offspring compared to the CTL group, while in female offspring, RSV partly diminished the effect on H3K9 di-methylation, but completely diminished the effect on H3K27 tri-methylation (see detailed statistical information in Additional file 1: Data S2). Our results indicate that postnatal RSV administration diminishes prenatal levonorgestrel exposure-induced methylation of DNA and histone on the ERβ promoter in the amygdala.

### Postnatal resveratrol administration ameliorates prenatal norethindrone exposure-induced oxidative stress and dysfunction of mitochondria and lipid metabolism

**Fig. 4:**
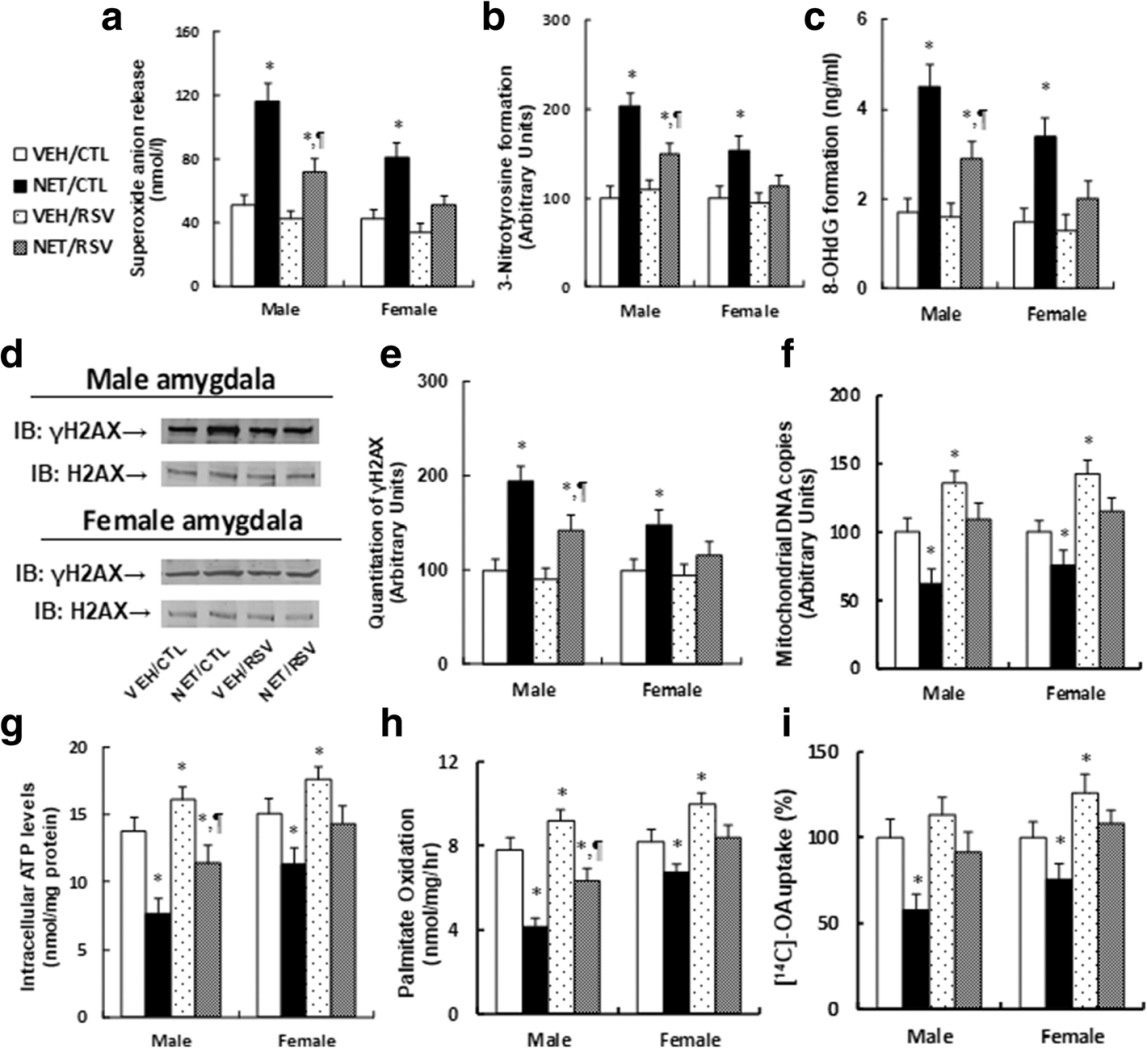
Postnatal resveratrol treatment ameliorates prenatal norethindrone exposure-induced oxidative stress, dysfunction of mitochondria, and lipid metabolism. **a–h** The amygdala tissues were isolated from 10-week-old male/female offspring for further analysis. **a** In vivo superoxide anion release, *n* = 5. **b** Quantitation of 3-nitrotyrosine (3-NT) formation, *n* = 5. **c** 8-OHdG formation, *n* = 5. **d** Representative γH2AX western blotting band for both male (upper panel) and female (down panel) offspring. **e** Quantitation of γH2AX formation for **d**, *n* = 5. **f** Mitochondrial DNA copies, *n* = 4. **g** Intracellular ATP levels, *n* = 5. **h** The in vivo palmitate oxidation rate, *n* = 5. **i** The amygdala neurons were isolated from 10-week-old male/female offspring for in vitro ^14^C-OA fatty acid uptake, *n* = 5. *, *P* < 0.05, vs VEH/CTL group; ¶, *P* < 0.05, vs NET/CTL group. Results are expressed as mean ± SEM

We evaluated the effect of RSV treatment on prenatal NET exposure-induced ERβ suppression and the subsequent molecular consequences in the amygdala, including ROS generation, DNA damage, mitochondrial function and lipid metabolism. We found that prenatal NET exposure significantly increased superoxide anion (O_2_^−^) release by 2.27-fold (in male) and 1.88-fold (in female) respectively (see Fig. 4a); it increased 3-nitrotyrosine formation by 2.03-fold (in male) and 1.54-fold (in female) respectively (see Fig. 4b); it increased 8-OHdG formation by 2.65-fold (in male) and 2.27-fold (in female) respectively (see Fig. 4c); it increased γH2AX formation by 1.94-fold (in male) and 1.48-fold (in female) respectively (see Fig. 4d, e); it decreased mitochondrial DNA copies by 38% (in male) and 24% (in female) respectively (see Fig. 4f); it decreased intracellular ATP level by 45% (in male) and 25% (in female) respectively (see Fig. 4g). Furthermore, it decreased in vivo fatty acid oxidation by 47% (in male) and 18% (in female) respectively (see Fig. 4h), and it decreased in vitro fatty acid uptake by 42% (in male) and 26% (in female) respectively (see Fig. 4i). RSV treatment partly reversed this effect in male offspring, while in female offspring, RSV completely diminished this effect. Furthermore, in VEH group, RSV treatment significantly increased mitochondrial function (see Fig. 4f, g) and in vivo fatty acid oxidation metabolism (see Fig. 4h) in both male and female offspring. On the other hand, RSV treatment increased in vitro fatty acid uptake (see Fig. 4i) in female offspring but not in male offspring from VEH group (see detailed statistical information in Additional file 1: Data S3). Our results indicate that RSV ameliorates prenatal NET exposure-induced oxidative stress and dysfunction of mitochondria and lipid metabolism, and that female offspring were less responsive to NET exposure than male offspring.

### Postnatal resveratrol treatment ameliorates prenatal norethindrone exposure-induced autism-like behavior

**Fig. 5:**
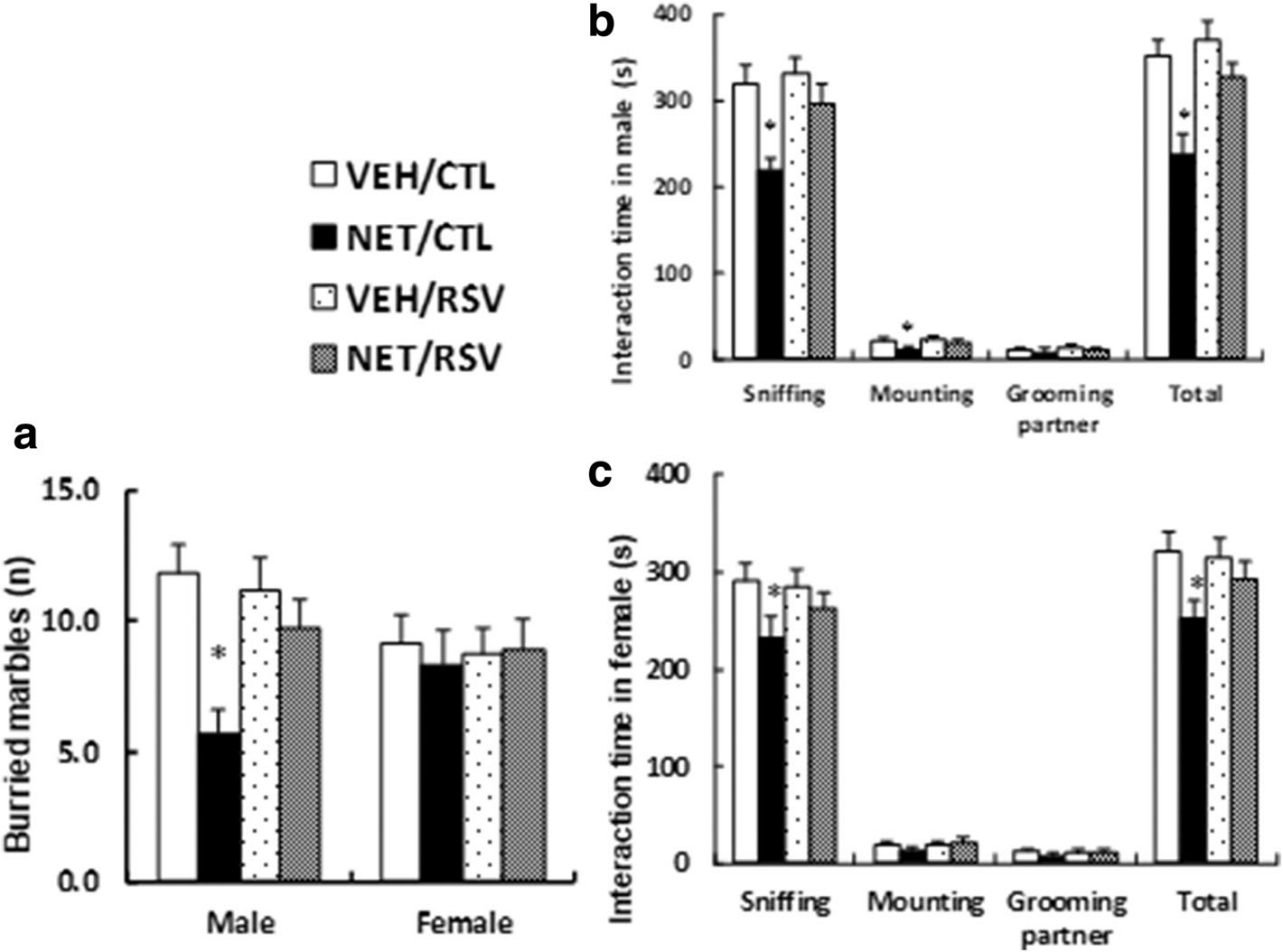
Postnatal resveratrol treatment ameliorates prenatal norethindrone exposure-induced autism-like behavior. Three-month-old pregnant dams were exposed to NET (20 μg levonorgestrel) or VEH (vehicle, 5% ethanol in organic sesame oil) by subcutaneous daily injection of 0.1 ml for 21 days until pup delivery. Both male and female offspring were then treated by either control (CTL) or resveratrol (RSV) for 4 weeks starting from 5 weeks old. Both male and female offspring were used for autism-like behavior tests at 10 weeks old. **a** Buried marble tests, *n* = 9. **b**, **c** Social interaction time in both male (**b**) and female (**c**) offspring, *n* = 9. *, *P* < 0.05, vs VEH/CTL group; ¶, *P* < 0.05, vs NET/CTL group. Results are expressed as mean ± SEM

We measured the effect of RSV on prenatal NET exposure-induced autism-like behavior. In male offspring, prenatal NET exposure decreased buried marbles by 52% (see Fig. 5a), while there was no difference in female offspring. We then evaluated the social interaction time. In male offspring, the NET treatment decreased by 32% in sniffing, 48% in mounting, no difference in grooming partner, and 33% in total social interaction (see Fig. 5b), while RSV treatment completely diminished this effect. In female offspring, the NET treatment decreased by 20% in sniffing, no difference in mounting or grooming partner, and 22% in total social interaction (see Fig. 5c). Furthermore, the RSV treatment completely diminished this effect in both male and female offspring (see detailed statistical information in Additional file 1: Data S4). Our results indicate that RSV completely reverses prenatal NET exposure-induced autism-like behavior and that male offspring seem more sensitive to NET-induced effect compared to female offspring.

### Postnatal resveratrol treatment ameliorates prenatal norethindrone exposure-induced autism-like behavior through ERβ activation

**Fig. 6:**
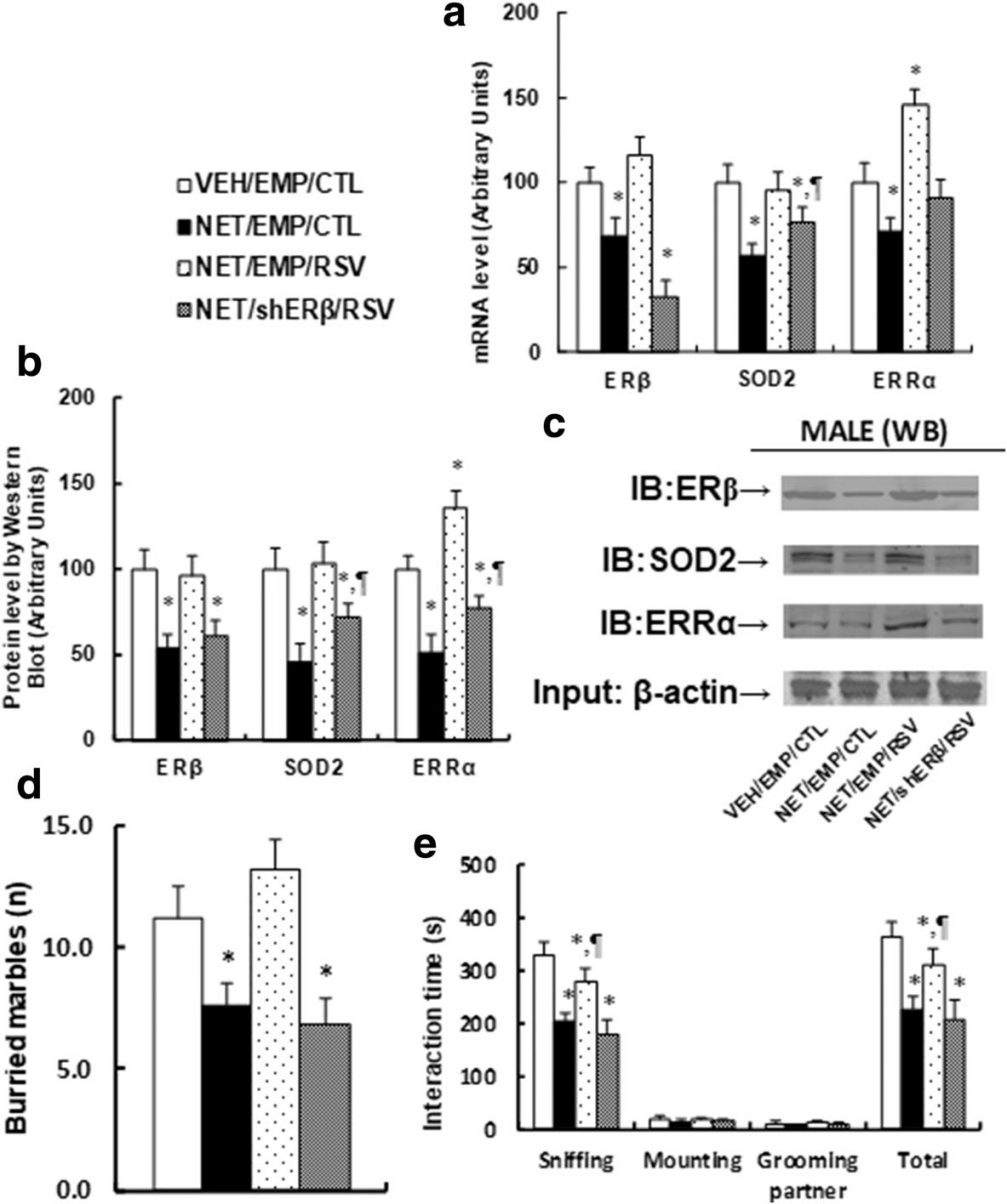
Postnatal resveratrol treatment ameliorates prenatal norethindrone exposure-induced autism-like behavior through ERβ activation. The 8-week-old male offspring from VEH or NET group received either empty (EMP) or ERβ knockdown (shERβ) lentivirus infusion and were treated by either control (CTL) or resveratrol (RSV) for 4 weeks, and the offspring were sacrificed at 13 weeks of age for further analysis. **a–c** The amygdala tissues were isolated from 13-week-old treated male offspring for gene expression analysis. **a** The mRNA levels for gene expression, *n* = 4. **b** The quantitation of protein levels, *n* = 5. **c** Representative bands for western blots. **d** Buried marble tests, *n* = 9. **e** Interaction time, *n* = 9. *, *P* < 0.05, vs VEH/EMP/CTL group; ¶, *P* < 0.05, vs NET/EMP/CTL group; #, *P* < 0.05, vs NET/EMP/RSV group. Results are expressed as mean ± SEM

Prenatal NET exposure treated male offspring received lentivirus infusion of shERβ shown in Fig. 1b. We first measured the gene expression of ERβ, SOD2, and ERRα. The results showed that prenatal NET exposure significantly decreased mRNA levels (see Fig. 6a) and protein levels (see Fig. 6b, c) of those genes. We then measured prenatal NET exposure-mediated molecular consequences in the amygdala. The results showed that postnatal resveratrol treatment ameliorates prenatal NET exposure-induced oxidative stress (see Additional file 1: Figure S3a, b), DNA damage (see Additional file 1: Figure S3c, e), dysfunction of mitochondria (see Additional file 1: Figure S3f, g), and lipid metabolism (see Additional file 1: Figure S3 h, i) through ERβ activation. RSV treatment completely reversed this suppression effect, while ERβ knockdown (shERβ) significantly diminished the effect of RSV (see Additional file 1: Figure S3). Next, we investigated the potential effect of RSV treatment and ERβ expression on prenatal NET exposure-induced autism-like behavior in male offspring. We found that prenatal NET exposure (NET/EMP/CTL) decreased buried marbles by 32% (see Fig. 6d) compared to the control group. RSV treatment completely reversed prenatal NET exposure-induced autism-like behavior, while ERβ knockdown (shERβ) completely diminished the effect of RSV. We also measured the social interaction time (see Fig. 6e). The results showed that prenatal NET exposure resulted in a 38% decrease in sniffing and a 37% decrease in total social interaction, while it showed no difference in mounting and grooming partner. RSV treatment partly diminished this effect, while ERβ knockdown (shERβ) completely diminished the effect of RSV (see detailed statistical information in Additional file 1: Data S5). Our results indicate that RSV ameliorates prenatal norethindrone exposure-induced autism-like behavior in offspring through ERβ activation.

### Prenatal resveratrol treatment prevents prenatal norethindrone exposure-induced autism-like behavior

**Fig. 7:**
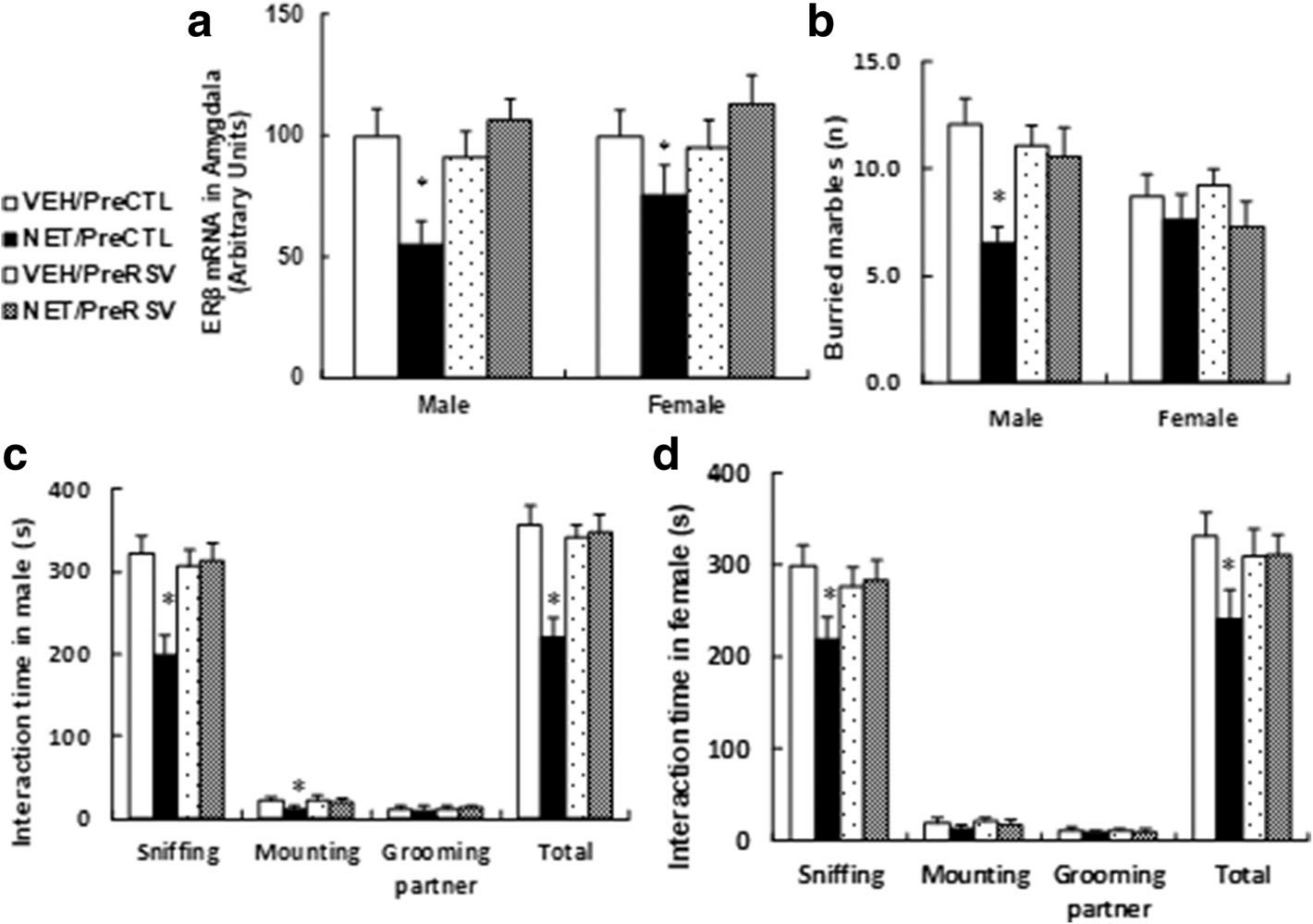
Prenatal resveratrol treatment prevents prenatal norethindrone exposure-induced autism-like behavior. Three-month old pregnant dams were exposed to NET (20 μg norethindrone) or VEH (vehicle only) by subcutaneous daily injection of 0.1 ml for 21 days until pup delivery. In addition, all the dams received either control (PreCTL) or resveratrol (PreRSV) treatment by oral administration at the same time. At 10 weeks of age, both male and female offspring were used for gene expression analysis and autism-like behavior testing. **a** The ERβ mRNA level, *n* = 4. **b** Buried marble tests, *n* = 9. **c, d** Interaction time for both male (**c**) and female (**d**) offspring, *n* = 9. *, *P* < 0.05, vs VEH/CTL group. Results are expressed as mean ± SEM

Three-month-old pregnant dams were exposed to either NET (20 μg norethindrone) or VEH (vehicle) for ~ 21 days during the whole pregnancy, and they also received either control (PreCTL) or resveratrol (PreRSV) prenatal treatment by oral administration. Both male and female offspring at 10 weeks of age were used for biomedical analysis and autism-like behavior testing. We first measured the ERβ gene expression (see Fig. 7a) and found that prenatal RSV treatment completely reversed prenatal NET exposure-induced ERβ suppression. We also measured the epigenetic changes with histone methylation on the ERβ promoter in both male (see Additional file 1: Figure S4a) and female offspring (see Additional file 1: Figure S4b). We found that prenatal RSV treatment completely normalized prenatal NET exposure-induced histone methylation, including H3K9me2 and H3K27me3. We also measured the oxidative stress, including superoxide anion (see Additional file 1: Figure S4c) and 8-OHdG formation (see Additional file 1: Figure S4d); mitochondria functions, including mitochondrial DNA copies (see Additional file 1: Figure S4e) and intracellular ATP level (see Additional file 1: Figure S4f); as well as lipid metabolism, including palmitate oxidation (see Additional file 1: Figure S4 g) and fatty acid uptake (see Additional file 1: Figure S4 h). It showed that prenatal RSV treatment completely (in female offspring) or partly (in male offspring) reversed NET-induced effect compared to control group. Finally, we measured autism-like behavior, including buried marble testing (see Fig. 7b) and social interaction time testing for both male (see Fig. 7c) and female offspring (see Fig. 7d). We found that prenatal RSV treatment completely reversed prenatal NET exposure-induced autism-like behavior in both male and female offspring (see detailed statistical information in Additional file 1: Data S6). Our results indicate that prenatal RSV treatment prevents prenatal NET exposure-induced autism-like behavior.

## Discussion

In this study, we show that prenatal progestin exposure decreases ERβ expression in the brain and induces autism-like behavior in offspring. Oral administration of RSV reverses prenatal progestin exposure-induced ERβ suppression by demethylation of DNA and histone on the ERβ promoter in the amygdala in offspring. RSV-mediated ERβ activation then upregulates the expression of SOD2 and ERRα and minimizes the oxidative stress and dysfunction of mitochondria [6] and lipid metabolism, subsequently ameliorating prenatal progestin exposure-induced autism-like behavior. In addition, prenatal resveratrol treatment prevents prenatal progestin exposure-induced autism-like behavior in offspring.

### Association of progestin and ASD development

Natural progesterone or synthetic progestins, such as hydroxyprogesterone caproate (OHPC), have been used to reduce the incidence of preterm birth [44], where there is a signal for embryo-fetal toxicity associated with OHPC in the two largest clinical trials [45]. Progestin is the major component in hormone replacement therapy (HRT) and combined oral contraceptive (COC), and taking oral contraceptives when pregnancy begins is a strong risk factor for ASD development [8]. Also, progestin concentrations found in water can suppress ERβ expression and affect brain functions [21, 46]. Our current data show that prenatal progestin exposure suppresses ERβ expression in the amygdala [47] and subsequently contributes to ASD development [9]. This consideration may potentially apply to humans, as prenatal progestin exposure may be associated with ASD development via directly or indirectly taking progestin compounds, which includes contraceptive pills, progesterone/progestin pills to prevent threatened abortion, and COC-contaminated drinking water and seafood during pregnancy [21].

### Resveratrol-mediated demethylation of DNA and histone on the ERβ promoter

It has been reported that RSV inhibits DNA methyltransferases (DNMTs) [48] and subsequently decreases DNA methylation [49] and histone methylation [50, 51]. We show that RSV in vivo treatment significantly decreases both DNA methylation and histone methylation on the ERβ promoter, and subsequently increases ERβ expression. This is the first time we report the potential neuroprotective mechanism of RSV through RSV-mediated demethylation with subsequent gene activation. Furthermore, we show that RSV treatment can only demethylate prenatal NET exposure-mediated hypermethylation on both DNA and histone, while it has little effect on basal methylation in the prenatal VEH exposure group. This suggests that RSV-mediated demethylation is not only due to RSV-mediated DNMT inhibition, but rather that some other indirect factors may also be involved [52].

### Resveratrol-mediated ERβ activation

We have previously reported that ERβ expression is regulated by SIRT1 through complexes of SIRT1-PPARγ/RXR-p300 on the ERβ promoter [14], and RSV has been reported to be able to activate SIRT1 either directly [53] or indirectly through AMPK activation [54]. We suppose that resveratrol treatment should be able to activate SIRT1 and subsequently upregulate ERβ expression, while our results show that RSV treatment significantly increases ERβ expression, but neither SIRT1 expression nor activity changes in the amygdala, suggesting that a SIRT1-independent ERβ activating mechanism is required for RSV-mediated ERβ activation. Our further investigation shows that RSV treatment in vivo significantly alters DNA methylation and epigenetic changes with demethylation on the ERβ promoter in the amygdala, which subsequently activates ERβ expression with ameliorated autism-like behavior. This is the first time we report the potential mechanism for RSV-mediated gene activation through demethylation of either DNA or histone in the nervous system, which sheds a light on rescuing ASD symptoms through resveratrol treatment.

### Resveratrol-mediated activation of SOD2 and ERRα

Our results show that resveratrol (RSV) treatment reverses prenatal NET exposure-induced methylation of DNA and histone on the ERβ promoter and subsequently reverses prenatal NET exposure-induced ERβ suppression, but it does not change the basal DNA and histone methylation level and subsequent basal ERβ expression. On the other hand, the basal expression level of SOD2 and ERRα increases significantly in response to RSV treatment in not only the amygdala, but also in the hypothalamus and hippocampus; this kind of gene activation is not regulated by ERβ as reported previously [11, 12] since the basal ERβ expression has no change in response to RSV treatment. Also, the in vitro cell culture experiments show that RSV treatment significantly increases the expression of SOD2 and ERRα, and female offspring seem more responsive to RSV treatment compared to male offspring. This can be explained because the female offspring have higher basal ERβ expression compared to male offspring, or because RSV can directly activate SOD2 and ERRα expression through another pathway, such as PGC1α [53]. Furthermore, in female rats, RSV increased expression of both SOD2 and ERRα, even in rats that were not exposed to NET, suggesting a more general effect. Our results indicate that another factor other than ERβ may also be involved in regulating the gene activation of SOD2 and ERRα. This can be explained by RSV-mediated PGC-1α activation, as it has been reported that RSV activates PGC-1α either by SIRT1 [53] or AMPK (54)-mediated indirect activation or by direct deacetylation of PGC-1α [55], and the activated PGC-1α then upregulates the expression of ERRα [56, 57] and SOD2 [58].

### Limitations

This study has several limitations. In animal behavioral tests, the marble burying test and the social interaction time were used for the evaluation of autism-like behavior, while these tests do not really reflect adequate tests of this syndrome. The marble burying test has been used to describe anxiety and repetitive behavior, but not clearly presented as a test for autism. A more common test for autism should be the social preference test and the ultrasonic vocalization test. In addition, during the animal treatment, prenatal exposure of either progestin or resveratrol was used throughout the whole pregnancy period (21 days), while this rarely happens in the human being. Usually, the first trimester during the pregnancy is considered as the most sensitive period for the prenatal exposure of risk factors; in this case, prenatal exposure of progestin or resveratrol during the first 7 days of pregnancy in dams may be more reasonable for this treatment. On the other hand, we were using higher doses of resveratrol and progestin for the treatment of pregnant dams in this study in order to achieve a significant effect in animals to mimic the long-term exposure of lower doses in humans, and this may be a potential limitation for human application.

## Conclusions

Our results indicate that prenatal progestin exposure is a strong risk factor for autism-like behavior. Many potential clinical progestin applications, including oral contraceptive pills, preterm birth drugs, and progestin-contaminated drinking water or seafood, may be risk factors for potential ASD development. In addition, resveratrol may be a good candidate for rescuing and preventing ASD symptoms in humans through ERβ upregulation.

## Additional file

Additional file 1: **Table S1.** Sequences of primers for the real time quantitative PCR (qPCR). **Figure S1.** Postnatal resveratrol treatment increases expression of SOD2 and ERRα, while it has no effect on the expression of ERβ and SIRT1 in the hypothalamus and hippocampus of prenatal norethindrone exposed offspring. **Figure S2.** Both resveratrol and norethindrone treatment do not change the expression and activity of SIRT1 in the amygdala. **Figure S3.** Postnatal resveratrol treatment ameliorates prenatal norethindrone exposure-induced oxidative stress, dysfunction of mitochondria and lipid metabolism through ERβ activation. **Figure S4.** Prenatal resveratrol treatment prevents prenatal norethindrone exposure-induced epigenetic changes, oxidative stress, and the dysfunction of mitochondria and lipid metabolism. Data S1. Statistical details for Fig. 2. Data S2. Statistical details for Fig. 3. Data S3. Statistical details for Fig. 4. Data S4. Statistical details for Fig. 5. Data S5. Statistical details for Fig. 6. Data S6. Statistical details for Fig. 7. (DOCX 402 kb)

## Abbreviations

ASD: Autism spectrum disorder
ChIP: Chromatin immunoprecipitation
COC: Combined oral contraceptive
E2: 17β-estradiol
EMP: Empty control
ERRα: Estrogen-related receptor α
ERβ: Estrogen receptor β
HRT: Hormone replacement therapy
LNG: Levonorgestrel
MBT: Marbles burying test
MPA: Medroxyprogesterone acetate
NEN: Norethynodrel
NES: Nestorone
NET: Norethindrone
NETA: Norethindrone acetate
NGM: Norgestimate
OHPC: Hydroxyprogesterone caproate
P4: Progesterone
RSV: Resveratrol
SI: Social interaction
SOD2: Mitochondrial superoxide dismutase
VEH: Vehicle control
