# Supplementary material for: Resveratrol ameliorates prenatal progestin exposure-induced autism-like behavior through ERβ activation
Source: Mol Autism. 2018 Aug 2;9:43. doi: 10.1186/s13229-018-0225-5 (PMC6090838; doi:10.1186/s13229-018-0225-5)
Supplement: Supplementary file 1 — Table S1. Sequences of primers for the real time quantitative PCR (qPCR). Figure S1. Postnatal resveratrol treatment increases expression of SOD2 and ERRα, while it has no effect on the expression of ERβ and SIRT1 in the hypothalamus and hippocampus of prenatal norethindrone exposed offspring. Figure S2. Both resveratrol and norethindrone treatment do not change the expression and activity of SIRT1 in the amygdala. Figure S3. Postnatal resveratrol treatment ameliorates prenatal norethindrone exposure-induced oxidative stress, dysfunction of mitochondria and lipid metabolism through ERβ activation. Figure S4. Prenatal resveratrol treatment prevents prenatal norethindrone exposure-induced epigenetic changes, oxidative stress, and the dysfunction of mitochondria and lipid metabolism. Data S1. Statistical details for Fig. 2. Data S2. Statistical details for Fig. 3. Data S3. Statistical details for Fig. 4. Data S4. Statistical details for Fig. 5. Data S5. Statistical details for Fig. 6. Data S6. Statistical details for Fig. 7. (DOCX 402 kb) [file 13229_2018_225_MOESM1_ESM.docx]

**Resveratrol Ameliorates Prenatal Progestin Exposure-Induced Autism-Like Behavior through ERβ Activation**

**Additional file 1**

MATERIALS AND METHODS

**Materials.** The antibodies for ERRα (ab37438), H2AX (ab20669) and γH2AX (ab2893), H3K9me2 (ab1220), H3K9me3 (ab8898) and H3K27me3 (ab6002) were obtained from Abcam. Antibodies for β-actin (sc-47778), ERβ (sc-137381), and SOD2 (sc-30080) were obtained from Santa Cruz Biotechnology. 17β-estradiol (E2, #E2758); progesterone (P4, #P0130); levonorgestrel (LNG, #1362602); medroxyprogesterone acetate (MPA, #1378001); nestorone (NES, # SML0550); norethindrone (NET, #1469005); norethindrone acetate (NETA, #1470004); norgestimate (NGM, # 1471914); hydroxyprogesterone caproate (OHPC, #1329006) and resveratrol (RSV, #R5010) were obtained from Sigma. Norethynodrel (NEN, #E4600-000) was obtained from Steraloids. 3-nitrotyrosine (3-NT) was measured using the 3-Nitrotyrosine ELISA Kit (ab116691 from Abcam) per manufacturers’ instructions. The mitochondrial fraction was isolated using a Pierce Mitochondria Isolation Kit (Pierce Biotechnology) per manufacturers’ instructions. Nuclear extracts were prepared using the NE-PER Nuclear and Cytoplasmic Extraction Reagents Kit (Pierce Biotechnology). Protein concentration was measured using the Coomassie Protein Assay Kit (Pierce Biotechnology) (1).

**Generation of Rat ERβ shRNA** **lentivirus**. According to our preliminary data from *in vitro* cell culture experiments, the following sequence was confirmed as the most effective in knockdown rat ERβ: 5’- GGT CAT GTG AAG GAT GTA AGG -3’. The shRNA template for ERβ or scrambled was designed (sense strand + loop + antisense strand) and the related double strand DNA (dsDNA) was synthesized and annealed. They were then inserted into the pLVX-shRNA1 vector (from Clontech) using BamH1/EcoR1 restriction sites. The Scrambled (CTL) or shERβ lentivirus was then expressed through Lenti-X™ shRNA Expression Systems (from Clontech) per manufacturers’ instructions (1).

**In vivo rat experiments.** The animal protocol conformed to US NIH guidelines (Guide for the Care and Use of Laboratory Animals, No. 85-23, revised 1996), and was reviewed and approved by the Institutional Animal Care and Use Committee from Wuhan University (1).

Protocol 1 for postnatal treatment of resveratrol. 3-month old female Sprague Dawley rats were caged with proven males, and the verified pregnant dams were randomly assigned to either 20mg of progestin (such as norethindrone, NET) or VEH (vehicle group that received the same volume of vehicle). Drugs were suspended in 5% ethanol in organic sesame oil and 0.1ml were given daily through subcutaneous injection at the nape starting from day 1 until pup delivery for ~21 days. The male and female offspring were separated from the dams on day 21, and then at 5 weeks old, the offspring from either VEH or progestin prenatal treatment were randomly divided into two groups, a resveratrol (RSV) group and a control (CTL) group. Rats in the RSV group were orally administered (by gavage) 20 mg/kg of RVS suspended in 10 g/L carboxymethylcellulose every day for 4 weeks (28 days). Those in the CTL group were administered 10 mL/kg of 10 g/L carboxymethylcellulose during the same period. At 10 weeks old, treated offspring were used for autism-like behavior testing, or were sacrificed for further experiments and biomedical analysis (1); see schematic details in Fig 1a.

Protocol 2 for postnatal treatment of resveratrol with infusion of shERβ lentivirus. The male offspring (8 weeks old) from the VEH and NET group in Protocol 1 were anesthetized with a mixture of ketamine (90 mg/kg) and xylazine (2.7 mg/kg) and implanted with a guide cannula targeting the amygdala (26 gauge; Plastics One). The following coordinates were chosen for the amygdala: −2.0mm posterior to bregma, ±4.2mm from the midline, and −7.2 mm from the skull surface on which it was based. Cannula was attached to the skull with dental acrylic and jeweler’s screws and closed with an obturator (2). An osmotic minipump (Alzet model 2002; flow rate 0.5 μl/h; Cupertino, CA) connected to a 26-gauge internal cannula that extended 1 mm below the guide was implanted and used to deliver either ERβ knockdown (shERβ), or empty (EMP) lentivirus. Vehicle consisting of artificial cerebrospinal fluid (aCSF; 140 mM NaCl, 3 mM KCl, 1.2 mM Na_2_HPO_4_, 1 mM MgCl_2_, 0.27 mM NaH_2_PO_4_, 1.2 mMCaCl_2_, and 7.2 mM dextrose, pH 7.4) was used for the infusion of the lentivirus. Infusion (flow rate 0.5 µl/h) began immediately after placement of the minipump. 0.5μl of total 2×10^3^ cfu of lentivirus was infused for 1 hour (1). The experimental rats were separated into 4 groups (12 per group) at 9 weeks old. Group 1: VEH offspring with empty control lentivirus infusion plus oral administration of carboxymethylcellulose control treatment for 4 weeks (VEH/EMP/CTL); Group 2: NET offspring with empty control lentivirus infusion plus oral administration of carboxymethylcellulose control treatment for 4 weeks (NET/EMP/CTL); Group 3: NET offspring with empty control lentivirus infusion plus oral administration of resveratrol (RSV) treatment for 4 weeks (NET/EMP/RSV); Group 4: NET offspring with ERβ knockdown lentivirus infusion plus oral administration (by gavage) of RSV treatment for 4 weeks (VEH/shERβ/RSV). At 14 weeks old, the offspring were used for behavior testing followed by biomedical analysis (1); see schematic details in Fig 1b.

Protocol 3 for prenatal treatment of resveratrol. 3-month old verified pregnant dams were randomly assigned to the following 4 groups: Group 1: The rats received VEH (5% ethanol in organic sesame oil and 0.1ml were given daily through subcutaneous injection at the nape) plus control (CTL) group with oral administration of 10 mL/kg of 10 g/L carboxymethylcellulose (VEH/PreCTL); Group 2: Rats received 20μg of progestin (such as norethindrone, NET) with 5% ethanol in organic sesame oil plus) plus CTL group (NET/PreCTL); Group 3: The rats received VEH treatment plus resveratrol (RSV) group with oral administration (by gavage) of 20 mg/kg of resveratrol suspended in 10 g/L carboxymethylcellulose (VEH/PreRSV); Group 4: The rats received progestin (such as NET) group plus RSV group (NET/PreRSV). The rats received the above treatment starting from day 1 until pup delivery for ~21 days. The male and female offspring were separated from the dams on day 21 and fed until 10 weeks old for autism-like behavior testing and other biomedical analysis (1), see schematic details in Fig 1c.

**Animal behavior test.** The animal behavior test of offspring was carried out at 10 weeks of age. Female offspring were tested in the diestrus phase, which was confirmed by vaginal smears. Autism-like behavior was evaluated using the marbles burying test (MBT) and social interaction (SI) test.

Marbles burying test (MBT). In brief, each rat is placed in a clean cage (35 × 23 × 19 cm^3^) filled with wood chip bedding to a depth of 5 cm containing 20 colored glass marbles (1cm diameter) placed in a 5×4 arrangement. The number of marbles buried (>50% covered by bedding material) in 30 minutes was hand-scored by the experimenter (1, 3, 4).

Social interaction (SI) test. In short, the subjects (Test and Stranger) were separately habituated to the arena for 5 min before the test. During each test, the rats were placed into the apparatus over a period of 20 min and the time spent following, mounting, grooming, and sniffing any body parts of the other rat was taken as an indicator of social engagement, and the social interaction time was calculated and analyzed using EthoVision XT animal tracking software (Noldus, USA) (5). The animal used as the “Stranger” was used only once, and was a Sprague Dawley rat of the same gender, weight, and age, with no previous contact with the test rats (1, 3, 4).

**In vitro primary culture of amygdala neurons**. 10-week old offspring were used for the preparation of primary amygdala neurons. Amygdala tissues were dissected from rat offspring that were humanely sacrificed through cervical dislocation. Tissues were treated with 0.05% trypsin EDTA for 15 min at 37°C. Trypsin EDTA was replaced with soybean trypsin inhibitor (Sigma) for 5 min at 37°C to stop the reaction. This was then replaced with supplemented Neurobasal A (Invitrogen) followed by mechanical dissociation. Cells were then resuspended in culture media, including Neurobasal A, B27, 1×GlutaMAX and 100 U/ml Pen/Strep (from Invitrogen), and then the cells were incubated at 37°C, 5% CO_2_ (6). The isolated amygdala neurons were used for the analysis of DNA methylation, epigenetic changes by ChIP assay on the ERβ promoter, and in vitro fatty acid lipid uptake (1).

**RT reaction and real-time quantitative PCR.** Total RNA from the amygdala was extracted using the RNeasy Micro Kit (Qiagen), and the RNA was reverse transcribed using an Omniscript RT kit (Qiagen). All the primers were designed using Primer 3 Plus software with the Tm at 60°C, primer size of 21bp, and the product length in the range of 140-160bp (see Table S1). The primers were validated with the amplification efficiency in the range of 1.9-2.1, and the amplified products were confirmed with agarose gel. The real-time quantitative PCR was run on iCycleriQ (Bio-Rad) with the Quantitect SYBR green PCR kit (Qiagen). The PCR was performed by denaturation at 95°C for 8 min, followed by 45 cycles of denaturation at 95°C, annealing at 60°C, and extension at 72°C for 10s, respectively. 1 µl of each cDNA was used to measure target genes. The β-actin was used as the housekeeping gene for transcript normalization, and the mean values were used to calculate relative transcript levels with the ^ΔΔ^CT method per instructions from Qiagen. In brief, the amplified transcripts were quantified by the comparative threshold cycle method using β-actin as a normalizer. Fold changes in gene mRNA expression were calculated as 2^−ΔΔCT^ with CT = threshold cycle, ΔCT=CT(target gene)-CT(β-actin), and the ΔΔCT =ΔCT(experimental)-ΔCT (reference) (7).

**Western Blotting.** The amygdala tissues were lysed in an ice-cold lysis buffer (0.137M NaCl, 2mM EDTA, 10% glycerol, 1% NP-40, 20mM Tris base, pH 8.0) with protease inhibitor cocktail (Sigma). The proteins were separated in 10% SDS-PAGE and further transferred to the PVDF membrane. The membrane was incubated with appropriate antibodies, washed and incubated with HRP-labeled secondary antibodies, and then the blots were visualized using the ECL+plus Western Blotting Detection System (Amersham). The blots were quantitated by IMAGEQUANT, and the results were normalized by β-actin (7).

**SIRT1 activity assay**. The SIRT1 deacetylase activity was evaluated in nuclear extract from MECs using a SIRT1 Fluorometric Drug Discovery Kit (Cat #: BML-AK555, Enzo Life Sciences) according to manufacturers’ instructions. Fluorescent intensity was measured using a FLx800 microplate fluorescence reader (Bio-Tek). No enzyme and Time 0 negative controls were generated by incubating developer II solution with 2mM nicotinamide before mixing the substrates with or without samples. SIRT1 activity was calculated with the corrected arbitrary fluorescence units of the tested samples to No-enzyme control and expressed as fluorescent units relative to the control (8).

**In vivo superoxide anion (O_2_^.-^) release**. Superoxide anion release from the amygdala tissue was determined by a luminol-EDTA-Fe enhanced chemiluminescence (CL) system supplemented with DMSO-TBAC (Dimethyl sulfoxide-tetrabutyl-ammonium chloride) solution for extraction of released O_2_^.-^ from tissues, as described previously. The superoxide levels were calculated from the standard curve generated by the xanthine/xanthine oxidase reaction (9).

**Measurement of DNA breaks.** The 8-OHdG formation was measured using an OxiSelect™ Oxidative DNA Damage ELISA Kit (Cat No. STA320, from Cell Biolabs Inc.) per manufacturers’ instructions. The formation of γH2AX was measured from nuclear extracts by western blotting using H2AX as the input control (1).

**Evaluation of mitochondrial function.**

*Mitochondrial DNA copies.* The genomic DNA was extracted from the amygdala tissue using a QIAamp DNA Mini Kit (Qiagen) and the mitochondrial DNA was extracted using the REPLI-g Mitochondrial DNA Kit (Qiagen). The purified DNA was used for the analysis of genomic β-actin (marker of the nuclear gene) and ATP6 (ATP synthase F0 subunit 6, marker of the mitochondrial gene) respectively using the qPCR method mentioned above. The primers for genomic β-actin: forward 5’-acc aca gct gag agg gaa atc -3’ and reverse 5’- att gcc gat agt gat gac ctg-3’. The primers for ATP6: forward 5’- tag ggc ttc ttc ccc ata cat -3’ and reverse 5’- tta gtg aga tgg ggg ttc ctt-3’. The mitochondrial DNA copies were obtained from relative ATP6 copies that were normalized by β-actin copies using the ^ΔΔ^CT method (1).

*Intracellular ATP level.* The intracellular ATP level was determined using the luciferin/luciferase-induced bioluminescence system. An ATP standard curve was generated at concentrations of 10^-12^-10^-3^M. Intracellular ATP levels were calculated and expressed as nmol/mg protein (1, 9).

**DNA methylation analysis.** We developed a real-time PCR based method for methylation specific PCR (MSP) analysis on the rat ERβ promoter according to the previously described method with some modifications (10-12). The rat genomic DNA from the amygdala was extracted and purified, and then treated by bisulfite modification using the EpiJET Bisulfite Conversion Kit (#K1461, Fisher). The modified DNA was then amplified using methylated and unmethylated primers for MSP that were designed using the Methprimer software: (<http://www.urogene.org/cgi-bin/methprimer/methprimer.cgi>) with the below details: Methylated primer Forward 5’- TTT TTT TTA GGT TTT TAA AAG ACG T-3’, Reverse 5’- ATA CCA ATA ACA ACA CCA ACC G -3’; Unmethylated primer Forward 5’- TTT TTT TTA GGT TTT TAA AAG ATG T -3’; Reverse 5’- AAT ACC AAT AAC AAC ACC AAC CAC T -3’. The product size: 194bp (methylated) & 195bp (unmethylated); CpG island size: 134bp; Tm: 68-70°C. The final methylation readout was normalized by unmethylated input PCR, the PCR products were confirmed by electrophorese using 2% agarose gel, and the DNA bands were imaged (1).

**Chromatin Immunoprecipitation (ChIP).** Cells were washed and crosslinked using 1% formaldehyde for 20 min and terminated by 0.1M glycine. Cell lysates were sonicated and centrifuged. 500µg of protein were pre-cleared by BSA/salmon sperm DNA with preimmune IgG and a slurry of Protein A Agarose beads. Immunoprecipitations were performed with the indicated antibodies, BSA/salmon sperm DNA and a 50% slurry of Protein A agarose beads. Input and immunoprecipitates were washed and eluted, then incubated with 0.2mg/ml Proteinase K for 2h at 42˚C, followed by 6h at 65˚C to reverse the formaldehyde crosslinking. DNA fragments were recovered by phenol/chloroform extraction and ethanol precipitation. A 140bp fragment in the range of -200~0 from the transcription start site on the rat ERβ promoter was amplified by real-time PCR (qPCR) using the below primers: forward 5’- ggg tgt ccc tag tgg atg act -3’ and reverse 5’- aaa aga gtg tgg gag ggt agc -3’ (1).

**Evaluation of fatty acid metabolism**

*In vitro lipid transport assay*. Primary amygdala neurons were seeded in a 12-well plate and grew until they were 80% confluent. After treatment, 0.5mCi well^-1^ of ^14^C-oleic acid (OA) from PerkinElmer was added. After 4h of incubation, the cells were washed and harvested, and the total radioactivity was quantitated by scintillation counting (1, 13).

*Rate of fatty acid oxidation from tissues*. The fatty acid oxidation (FAO) rate was measured by evaluation of palmitate oxidation using published methods with minor modifications (14, 15). In brief, the amygdala tissue was homogenized, and 30μl of tissue homogenate were then incubated in 370μl of DMEM containing 0.5% BSA/0.2mM palmitate/0.5μCi/mL 1-^14^C-palmitate at 37°C for 2h. The incubation was stopped by the injection of 0.2 ml of 40% perchloric acid into the tube to acidify the medium and liberate the CO_2_. The CO_2_ was trapped by a filter paper saturated with 20μL of 1M NaOH located on the top of the cap. After overnight isotopic equilibration at room temperature, the filter was removed, and the trapped ^14^CO_2_ and ^14^C acid-soluble products generated by the oxidation of [^14^C] palmitate were counted to calculate total palmitate oxidation. The protein concentrations were measured and the results were expressed as nmol per mg proteins per hour (nmol/mg/h) (1).

**Statistical analysis**. The data was given as mean ± SEM, and all the experiments were performed at least in quadruplicate unless indicated otherwise. In order to evaluate the effects of different treatments on the examined parameters in both males and females, the two-way analysis of variance (ANOVA) followed by the Bonferroni post hoc test was used; to evaluate the effects of treatments on the examined parameters in either males or females only, the one-way ANOVA followed by the Turkey−Kramer test was used to determine statistical significance of different groups using SPSS 22 software, and a *P* value of < 0.05 was considered significant (1).

REFERENCES

1. Zou Y, Lu Q, Zheng D, Chu Z, Liu Z, Chen H, Ruan Q, Ge X, Zhang Z, Wang X, et al. Prenatal levonorgestrel exposure induces autism-like behavior in offspring through ERbeta suppression in the amygdala. *Mol Autism.* 2017;8(46.

2. Hu M, Richard JE, Maliqueo M, Kokosar M, Fornes R, Benrick A, Jansson T, Ohlsson C, Wu X, Skibicka KP, et al. Maternal testosterone exposure increases anxiety-like behavior and impacts the limbic system in the offspring. *Proc Natl Acad Sci U S A.* 2015;112(46):14348-53.

3. Bahi A. Sustained lentiviral-mediated overexpression of microRNA124a in the dentate gyrus exacerbates anxiety- and autism-like behaviors associated with neonatal isolation in rats. *Behav Brain Res.* 2016;311(298-308.

4. Bahi A. Hippocampal BDNF overexpression or microR124a silencing reduces anxiety- and autism-like behaviors in rats. *Behav Brain Res.* 2017;326(281-90.

5. Mufford JT, Paetkau MJ, Flood NJ, Regev-Shoshani G, Miller CC, and Church JS. The development of a non-invasive behavioral model of thermal heat stress in laboratory mice (Mus musculus). *J Neurosci Methods.* 2016;268(189-95.

6. Hay CW, Shanley L, Davidson S, Cowie P, Lear M, McGuffin P, Riedel G, McEwan IJ, and MacKenzie A. Functional effects of polymorphisms on glucocorticoid receptor modulation of human anxiogenic substance-P gene promoter activity in primary amygdala neurones. *Psychoneuroendocrinology.* 2014;47(43-55.

7. Zhang H, Li L, Li M, Huang X, Xie W, Xiang W, and Yao P. Combination of betulinic acid and chidamide inhibits acute myeloid leukemia by suppression of the HIF1alpha pathway and generation of reactive oxygen species. *Oncotarget.* 2017;8(55):94743-58.

8. Hou X, Xu S, Maitland-Toolan KA, Sato K, Jiang B, Ido Y, Lan F, Walsh K, Wierzbicki M, Verbeuren TJ, et al. SIRT1 regulates hepatocyte lipid metabolism through activating AMP-activated protein kinase. *J Biol Chem.* 2008;283(29):20015-26.

9. Yao D, Shi W, Gou Y, Zhou X, Yee Aw T, Zhou Y, and Liu Z. Fatty acid-mediated intracellular iron translocation: a synergistic mechanism of oxidative injury. *Free Radic Biol Med.* 2005;39(10):1385-98.

10. Ogino S, Kawasaki T, Brahmandam M, Cantor M, Kirkner GJ, Spiegelman D, Makrigiorgos GM, Weisenberger DJ, Laird PW, Loda M, et al. Precision and performance characteristics of bisulfite conversion and real-time PCR (MethyLight) for quantitative DNA methylation analysis. *J Mol Diagn.* 2006;8(2):209-17.

11. Eads CA, Danenberg KD, Kawakami K, Saltz LB, Blake C, Shibata D, Danenberg PV, and Laird PW. MethyLight: a high-throughput assay to measure DNA methylation. *Nucleic Acids Res.* 2000;28(8):E32.

12. Nosho K, Irahara N, Shima K, Kure S, Kirkner GJ, Schernhammer ES, Hazra A, Hunter DJ, Quackenbush J, Spiegelman D, et al. Comprehensive biostatistical analysis of CpG island methylator phenotype in colorectal cancer using a large population-based sample. *PLoS ONE.* 2008;3(11):e3698.

13. Hagberg CE, Falkevall A, Wang X, Larsson E, Huusko J, Nilsson I, van Meeteren LA, Samen E, Lu L, Vanwildemeersch M, et al. Vascular endothelial growth factor B controls endothelial fatty acid uptake. *Nature.* 2010;464(7290):917-21.

14. Taib B, Bouyakdan K, Hryhorczuk C, Rodaros D, Fulton S, and Alquier T. Glucose regulates hypothalamic long-chain fatty acid metabolism via AMP-activated kinase (AMPK) in neurons and astrocytes. *J Biol Chem.* 2013;288(52):37216-29.

15. Huynh FK, Green MF, Koves TR, and Hirschey MD. Measurement of fatty acid oxidation rates in animal tissues and cell lines. *Methods Enzymol.* 2014;542(391-405.

**Table S1. Sequences of primers for the real time quantitative PCR (qPCR)**

| Gene | Species | Forward primer (5'→3') | Reverse primer (5'→3') |
| --- | --- | --- | --- |
| β-actin | Rat | ttccttcctgggtatggaatc | cttctgcatcctgtcagcaat |
| ERβ | Rat | tcagcatgaagtgcaaaaatg | ggttctgggagctctctttgt |
| ERRα | Rat | cagtgggaagctagtgctcag | ggacagctgtactcgatgctc |
| SOD2 | Rat | caactcaggttgctcttcagc | ctcaaaagacccaaagtcacg |
| SIRT1 | Rat | aagcgtcttgacggtaatcaa | aaacttggactctggcatgtg |

FIGURE S1

**Figure S1. Postnatal resveratrol treatment increases expression of SOD2 and ERRα, while it has no effect on the expression of ERβ and SIRT1 in the hypothalamus and hippocampus of prenatal norethindrone exposed offspring.** 3-month old pregnant dams were exposed to NET (20mg norethindrone), or VEH (vehicle, 5% ethanol in organic sesame oil) by subcutaneous daily injection of 0.1ml for 21 days until pup delivery. Both male and female offspring were then treated by either control (CTL) or resveratrol (RSV) for 4 weeks starting from 5 weeks old. The offspring were sacrificed at 10 weeks of age to isolate the hypothalamus and hippocampus tissues for further analysis. (a-c) The mRNA levels in hypothalamus for genes of ERβ (a), SOD2 (b), ERRα (c) and SIRT1 (d), n=5. (e-h) The mRNA levels in hippocampus for genes of ERβ (e), SOD2 (f), ERRα (g) and SIRT1 (h), n=5. Results are expressed as mean ± SEM.

FIGURE S2

**Figure S2. Both resveratrol and norethindrone treatment do not change the expression and activity of SIRT1 in the amygdala.** 3-month old pregnant dams were exposed to either NET (20mg norethindrone), or VEH (vehicle) for 21 days until pup delivery. Both male and female offspring were then treated with either control (CTL) or resveratrol (RSV, 20 mg/kg by oral administration) for 4 weeks starting at 5 weeks old. Rats were sacrificed at 10 weeks of age to isolate the amygdala for further analysis. (a) SIRT1 mRNA levels in amygdala, n=5. (b) SIRT1 activity assay, n=5. Results are expressed as mean ± SEM.

FIGURE S3

**Figure S3. Postnatal resveratrol treatment ameliorates prenatal norethindrone exposure-induced oxidative stress, dysfunction of mitochondria and lipid metabolism through ERβ activation.** The 8-week old male offspring from VEH or NET group received either empty (EMP) or ERβ knockdown (shERβ) lentivirus infusion, and treated by either control (CTL) or resveratrol (RSV) for 4 weeks, and the offspring were sacrificed at 13 weeks of age for further analysis. (a-h) The amygdala tissues were isolated from 13-week old treated male offspring for further analysis. (a) In vivo superoxide anion release, n=6. (b) Quantitation of 3-nitrotyrosine (3-NT) formation, n=5. (c) 8-OHdG formation, n=6. (d) Representative γH2AX western blotting band. (e) Quantitation of γH2AX formation, n=5. (f) Mitochondrial DNA copies, n=4. (g) Intracellular ATP levels, n=5. (h) The in vivo palmitate oxidation rate, n=5. (i) The amygdala neurons were isolated from 10-week old treated male offspring for in vitro ^14^C-OA fatty acid uptake, n=5. *, *P*<0.05, vs VEH/EMP/CTL group; ¶, *P*<0.05, vs NET/EMP/CTL group; #, *P*<0.05, vs NET/EMP/RSV group. Results are expressed as mean ± SEM.

FIGURE S4

**Figure S4. Prenatal resveratrol treatment prevents prenatal norethindrone exposure-induced epigenetic changes, oxidative stress, and the dysfunction of mitochondria and lipid metabolism.** 3-month old pregnant dams were exposed to NET (20μg norethindrone), or VEH (vehicle only) by subcutaneous daily injection of 0.1ml for 21 days until pup delivery. In addition, all the dams received either control (PreCTL) or resveratrol (PreRSV) treatment by oral administration at the same time. Both male and female offspring at 10 weeks of age were used for biomedical analysis and autism-like behavior testing. (a) ChIP analysis on the ERβ promoter in female amygdala neurons, n=5. (b) In vivo superoxide anion release, n=6. (c) 8-OHdG formation, n=5. (d) Mitochondrial DNA copies, n=4. (f) Intracellular ATP level, n=5. (g) The in vivo palmitate oxidation rate, n=5. (h) The amygdala neurons were isolated from 10-week old male/female offspring for in vitro ^14^C-OA fatty acid uptake, n=5. *, *P*<0.05, vs VEH/CTL group. Results are expressed as mean ± SEM.

**Data S1. Statistical details for Figure 2**

In Fig 2a, two-way ANOVA revealed a significant effect of sex, [F(1, 32)=4.364, P=0.042], and a significant effect on treatment [F(3,32)=4.698, P=0.026], and significant interaction [F(3,32)= 4.016, P=0.037]. Subsequent post hoc analysis revealed that in male, NET decreased ERβ expression by -51% vs. VEH group, p<0.01; and RSV increased ERβ expression by 216% vs. CTL group, p<0.01. In female, NET decreased ERβ expression by -31% vs. VEH group, p=0.034; and RSV increased ERβ expression by 154% vs. CTL group, p<0.01. The female had significant less response in NET treatment compared to male (p=0.026).

In Fig 2b, two-way ANOVA revealed a significant effect of sex, [F(1, 32)=4.256, P=0.039], and a significant effect on treatment [F(3,32)=4.968, P=0.027], and significant interaction [F(3,32)= 4.336, P=0.021]. Subsequent post hoc analysis revealed that in male, NET decreased SOD2 expression by -33% vs. VEH group, p<0.01; and RSV had no effect, P=0.361. In female, NET decreased SOD2 expression by -21% vs. VEH group, p=0.036; and RSV increased SOD2 expression by 159% vs. CTL group, p<0.01. The female had significant stronger response in RSV treatment compared to male (p<0.01).

In Fig 2c, two-way ANOVA revealed a significant effect of sex, [F(1, 32)=5.106, P=0.016], and a significant effect on treatment [F(3,32)=4.611, P=0.018], and significant interaction [F(3,32)=5.661, P=0.014]. Subsequent post hoc analysis revealed that in male, NET decreased ERRα expression by -28% vs. VEH group, p<0.01; and RSV increased ERRα expression by 152% vs. CTL group, p<0.01. In female, NET had no effect on ERRα expression vs. VEH group, p=0.98; and RSV increased ERRα expression by 157% vs. CTL group, p<0.01. The female had significant stronger response in RSV treatment compared to male (p<0.01).

In Fig 2f, for male offspring, one-way ANOVA revealed a significant effect on ERβ [F(3,19)= 9.652, P<0.001]; a significant effect on SOD2 [F(3,19)=11.312, P<0.001] and a significant effect on ERRα [F(3,19)=10.238, P<0.001]. Subsequent Turkey analysis revealed that NET decreased ERβ, SOD2 and ERRα expression by -55%, 61% and 28% respectively vs. VEH group, p<0.01); RSV increased SOD2 and ERRα by 132% and 140% respectively vs. CTL group, p<0.01).

In Fig 2g for female offspring, one-way ANOVA revealed a significant effect on ERβ [F(3,19)= 5.112, P=0.021]; a significant effect on SOD2 [F(3,19)=4.269, P=0.031], and significant effect on ERRα [F(3,19)=4.016, P=0.041]. Subsequent Turkey analysis revealed that NET decreased ERβ, SOD2 and ERRα expression by -35%, 28% and 21% respectively vs. VEH group, p<0.01); RSV increased ERRα expression by 130% (VEH group) and 125% (NET group) respectively vs. VEH/CTL group, p<0.01).

**Data S2. Statistical details for Figure 3**

In Fig 3b for ERβ promoter methylation, two-way ANOVA revealed no significant effect of sex, [F(1, 32)=2.102, P=0.075], a significant effect on treatment [F(3,32)=11.564, P<0.001], and a significant interaction [F(3,32)=5.611, P=0.016]. Subsequent post hoc analysis revealed that NET increased methylation by 168% (male offspring) and 154% (female offspring), respectively vs. VEH group, p<0.001.

In Fig 3c for male offspring, one-way ANOVA revealed a significant effect on H3K9me2 [F(3,19)=12.316, P<0.001], a significant effect on H3K27me3 [F(3,19)=14.259, P<0.001], and no significant effect on H3K9me3 [F(3,19)=1.274, P=0.215]. Subsequent Turkey analysis revealed that NET increased binding ability of H3k9me2 by 180% vs. VEH group, p<0.01); the NET/CTL and NET/RSV group increased H3K27me3 by 213% and 158% respectively vs. VEH/CTL group, p<0.01); and there was no effect on H3K27me3 (p=0.116).

In Fig 3d for female offspring, one-way ANOVA revealed a significant effect on H3K9me2 [F(3,19)=15.436, P<0.001], a significant effect on H3K27me3 [F(3,19)=13.644, P<0.001], and no significant effect on H3K9me3 [F(3,19)=1.211, P=0.119]. Subsequent Turkey analysis revealed that the NET/CTL and NET/RSV group increased binding ability of H3k9me2 by 214% and 135% respectively vs. VEH/CTL group, p<0.01); and the NET group increased H3K27me3 by 156% vs. VEH group, p<0.01); and there was no effect on H3K27me3 (p=0.128).

**Data S3. Statistical details for Figure 4**

In Fig 4a, two-way ANOVA revealed significant effect of sex, [F(1, 32)=6.211, P<0.01], and a significant effect on treatment [F(3,32)=10.237, P<0.001], and significant interaction [F(3,32)=5.101, P=0.018]. Subsequent post hoc analysis revealed that in male offspring, NET increased superoxide anion release by 228% vs. VEH group, p<0.01, and RSV partly reversed this effect, the NET/RSV group increased superoxide release by 141% vs. VEH/CTL group, p<0.01; In female offspring, NET increased superoxide anion release by 188% vs. VEH group, p<0.01); and RSV had no effect (p=0.791); Also, female offspring had significant less response in NET and RSV treatments compared to male offspring (p<0.01).

In Fig 4b, two-way ANOVA revealed significant effect of sex, [F(1, 32)=5.897, P<0.01], and a significant effect on treatment [F(3,32)=9.891, P<0.001], and a significant interaction [F(3,32)=4.694, P=0.021]. Subsequent post hoc analysis revealed that in male offspring, NET increased 3-NT formation by 203% vs. VEH group, p<0.01, and RSV partly reversed this effect, the NET/RSV group increased 3-NT formation by 149% vs. VEH/CTL group, p<0.01; In female offspring, NET increased 3-NT formation by 154% vs. VEH group, p<0.01; and RSV had no effect (p=0.883); Also, female offspring had significant less response in NET and RSV treatments compared to male offspring (p<0.01).

In Fig 4c, two-way ANOVA revealed significant effect of sex, [F(1, 32)=5.112, P<0.01], and a significant effect on treatment [F(3,32)=10.239, P<0.001], and a significant interaction [F(3,32)=4.325, P=0.036]. Subsequent post hoc analysis revealed that in male offspring, NET increased 8-OHdG formation by 265% vs. VEH group, p<0.01, and RSV partly reversed this effect, the NET/RSV group increased 8-OHdG formation by 171% vs. VEH/CTL group, p<0.01; In female offspring, NET increased 8-OHdG formation by 227% vs. VEH group, p<0.01; and RSV had no effect (p=0.914); Also, female offspring had significant less response in NET and RSV treatments compared to male offspring (p<0.01).

In Fig 4e, two-way ANOVA revealed significant effect of sex, [F(1,32)=4.213, P=0.023], and a significant effect on treatment [F(3,32)=11.316, P<0.001], and a significant interaction [F(3,32)=4.562, P=0.032]. Subsequent post hoc analysis revealed that in male offspring, NET increased γH2AX formation by 194% vs. VEH group, p<0.01, and RSV partly reversed this effect, the NET/RSV group increased γH2AX formation by 143% vs. VEH/CTL group, p<0.01; In female offspring, NET increased γH2AX formation by 148% vs. VEH group, p<0.01; and RSV had no effect (p=0.791); Also, female offspring had significant less response in NET and RSV treatments compared to male offspring (p<0.01).

In Fig 4f, two-way ANOVA revealed no effect of sex, [F(1,24)=2.133, P=0.0691], and a significant effect on treatment [F(3,24)=9.687, P<0.001], and no interaction [F(3,24)=1.895, P=0.071]. Subsequent post hoc analysis revealed that in male offspring, NET decreased mitochondrial DNA copies by 38% vs. VEH group, p<0.01, and RSV reversed this effect, the CTL/RSV group increased mitochondrial DNA copies by 136% vs. VEH/CTL group, p<0.01; In female offspring, NET decreased mitochondrial DNA copies by 24% vs. VEH group, p<0.01; and RSV reversed this effect, the CTL/RSV group increased mitochondrial DNA copies by 143% vs. VEH/CTL group, p<0.01.

In Fig 4g, two-way ANOVA revealed significant effect of sex, [F(1,32)=4.236, P=0.035], and a significant effect on treatment [F(3,32)=10.318, P<0.001], and no interaction [F(3,32)=2.364, P=0.068]. Subsequent post hoc analysis revealed that in male offspring, NET decreased intracellular ATP level by 45% vs. VEH group, p<0.01, and RSV partly reversed this effect, the CTL/RSV group increased intracellular ATP level by 118% vs. VEH/CTL group, p<0.05, and the NET/RSV group decreased intracellular ATP level by 17% vs. VEH/CTL group, p<0.05; In female offspring, NET decreased intracellular ATP level by 25% vs. VEH group, p<0.01; and RSV reversed this effect, the CTL/RSV group increased intracellular ATP level by 117% vs. VEH/CTL group, p<0.05.

In Fig 4h, two-way ANOVA revealed significant effect of sex, [F(1,32)=5.111, P=0.014], and a significant effect on treatment [F(3,32)=9.894, P<0.001], and no interaction [F(3,32)=3.164, P=0.056]. Subsequent post hoc analysis revealed that in male offspring, NET decreased palmitate oxidation by 47% vs. VEH group, p<0.01, and RSV partly reversed this effect, the CTL/RSV group increased palmitate oxidation by 118% vs. VEH/CTL group, p<0.05, and the NET/RSV group decreased palmitate oxidation by 19% vs. VEH/CTL group, p<0.05; In female offspring, NET decreased palmitate oxidation by 18% vs. VEH group, p<0.01; and RSV reversed this effect, the CTL/RSV group increased palmitate oxidation by 122% vs. VEH/CTL group, p<0.05.

In Fig 4i, two-way ANOVA revealed significant effect of sex, [F(1,32)=4.362, P=0.041], and a significant effect on treatment [F(3,32)=9.189, P<0.001], and no interaction [F(3,32)=3.281, P=0.062]. Subsequent post hoc analysis revealed that in male offspring, NET decreased fatty acid uptake by 42% vs. VEH group, p<0.01, and RSV reversed this effect. In female offspring, NET decreased fatty acid uptake by 26% vs. VEH group, p<0.01; and RSV reversed this effect, and the CTL/RSV group increased fatty acid uptake by 126% vs. VEH/CTL group, p<0.05.

**Data S4. Statistical details for Figure 5**

In Fig 5a, two-way ANOVA revealed significant effect of sex, [F(1, 64)=3.981, P=0.046], and a significant effect on treatment [F(3,64)=5.884, P<0.01], and a significant interaction [F(3,64)=4.265, P=0.038]. Subsequent post hoc analysis revealed that NET decreased buried marbles in male offspring by 52% vs. VEH group, p<0.01; and the female offspring had no effect in both NET and RSV treatments, p<0.01.

In Fig 5b for male offspring, one-way ANOVA revealed a significant effect on Sniffing [F(3,35)=7.125, P<0.01], Mounting F(3,35)=4.121, P=0.045], and Total interaction time F(3,35)=6.589, P<0.01], there was no significant effect on Grooming partner [F(3,35)=1.119, P=0.106]. Subsequent Turkey analysis revealed that NET decreased Sniffing, Mounting and Total interaction time by 32%, 48% and 33%, respectively, vs. VEH group, p<0.01. RSV completely reversed this effect, and there was no effect on Grooming partner (p=0.106).

In Fig 5c for female offspring, one-way ANOVA revealed a significant effect on Sniffing [F(3,35)=4.895, P=0.037] and Total interaction time F(3,35)=5.114, P=0.031], there was no significant effect on Mounting F(3,35)=2.131, P=0.067] and Grooming partner [F(3,35)=1.089, P=0.112]. Subsequent Turkey analysis revealed that NET decreased Sniffing and Total interaction time by 20% and 22%, respectively, vs. VEH group, p<0.01. RSV completely reversed this effect, and there was no effect on Mounting (p=0.091) and Grooming partner (p=0.106).

**Data S5. Statistical details for Figure 6**

In Fig 6a for mRNA expression, regarding the ERβ, one-way ANOVA revealed a significant effect [F(3,15)= 10.372, P<0.01]. Subsequent Turkey analysis revealed that the NET/EMP/CTL and NET/shERβ/RSV treatments decreased ERβ expression by 32% and 68%, respectively vs. VEH/EMP/CTL group, p<0.01; and there was no effect on NET/EMP/RSV treatment, p=0.819. Regarding the SOD2, one-way ANOVA revealed a significant effect [F(3,15)= 8.415, P<0.01]. Subsequent Turkey analysis revealed that NET/EMP/CTL and NET/shERβ/RSV treatments decreased SOD2 expression by 44% (p<0.01) and 24% (p<0.05), respectively vs. VEH/EMP/CTL group; and there was no effect on NET/EMP/RSV treatment, p=1.012. Regarding the ERRα, one-way ANOVA revealed a significant effect [F(3,15)= 9.246, P<0.01]. Subsequent Turkey analysis revealed that NET/EMP/CTL treatment decreased ERRα expression by 29% vs. VEH/EMP/CTL group, p<0.01; and NET/EMP/RSV treatment increased ERRα expression by 146% vs. VEH/EMP/CTL group, p<0.01; and there was no effect on NET/shERβ/RSV treatment, p=0.819.

In Fig 6b for protein expression, regarding the ERβ, one-way ANOVA revealed a significant effect [F(3,15)= 9.678, P<0.01]. Subsequent Turkey analysis revealed that NET/EMP/CTL and NET/shERβ/RSV treatments decreased ERβ expression by 46% and 39%, respectively vs. VEH/EMP/CTL group, p<0.01; and there was no effect on NET/EMP/RSV treatment, p=0.965. Regarding the SOD2, one-way ANOVA revealed a significant effect [F(3,15)= 9.325, P<0.01]. Subsequent Turkey analysis revealed that NET/EMP/CTL and NET/shERβ/RSV treatments decreased SOD2 expression by 54% (p<0.01) and 28% (p<0.05), respectively vs. VEH/EMP/CTL group; and there was no effect on NET/EMP/RSV treatment, p=0.915. Regarding the ERRα, one-way ANOVA revealed a significant effect [F(3,15)=10.196, P<0.01]. Subsequent Turkey analysis revealed that NET/EMP/CTL and NET/shERβ/RSV treatments decreased ERRα expression by 49% (p<0.01) and 23% (p<0.05), respectively vs. VEH/EMP/CTL group; and NET/EMP/RSV treatment increased ERRα expression by 136% vs. VEH/EMP/CTL group, p<0.01.

In Fig 6d for buried marbles testing, one-way ANOVA revealed a significant [F(3,35)= 9.635, P<0.01]. Subsequent Turkey analysis revealed that NET/EMP/CTL and NET/shERβ/RSV treatments decreased buried marbles by 32% and 39%, respectively vs. VEH/EMP/CTL group, p<0.01; and there was no effect on NET/EMP/RSV treatment, p=0.896.

In Fig 6e for social interaction time in male offspring, one-way ANOVA revealed a significant effect on Sniffing [F(3,35)=8.631, P<0.01] and Total interaction time F(3,35)=9.136, P<0.01], there was no significant effect on Mounting F(3,35)=1.415, P=0.071] and Grooming partner [F(3,35)=1.023, P=0.123]. Subsequent Turkey analysis revealed that NET/EMP/CTL, NET/EMP/RSV and NET/shERβ/RSV treatments decreased Sniffing by 38% (p<0.01), 15% (p<0.05) and 46% (p<0.01), respectively vs. VEH/EMP/CTL group; and decreased Total interaction time by 37% (p<0.01), 14% (p<0.05) and 43% (p<0.01), respectively vs. VEH/EMP/CTL group.

**Data S6. Statistical details for Figure 7**

In Fig 7a, two-way ANOVA revealed no significant effect of sex, [F(1, 32)=2.110, P=0.056], and a significant effect on treatment [F(3,32)=5.211, P=0.026], and significant interaction [F(3,32)=3.819, P=0.038]. Subsequent post hoc analysis revealed that NET/PreCTL decreased ERβ expression by 45% (in male, p<0.01) and 25% (in female, P<0.05), respectively vs. VEH/PreCTL group.

In Fig 7b for buried marbles, two-way ANOVA revealed a significant effect of sex, [F(1, 72)=3.698, P=0.041], and a significant effect on treatment [F(3,72)=5.667, P=0.021], and significant interaction [F(3,72)=3.965, P=0.043]. Subsequent post hoc analysis revealed that NET/PreCTL decreased buried marbles in male by 46% vs. VEH/PreCTL group, p<0.01; and there was no difference in female treatment, P=1.216.

In Fig 7c for male offspring, one-way ANOVA revealed a significant effect on Sniffing [F(3,35)=5.164, P=0.038], Mounting F(3,35)=4.362, P=0.046], and Total interaction time F(3,35)=5.264, P<0.034], there was no significant effect on Grooming partner [F(3,35)=1.231, P=0.097]. Subsequent Turkey analysis revealed that NET/PreCTL decreased Sniffing, Mounting and Total interaction time by 39%, 45% and 38%, respectively, vs. VEH/PreCTL group, p<0.01. RSV completely reversed this effect, and there was no effect on Grooming partner (p=0.089).

In Fig 7d for female offspring, one-way ANOVA revealed a significant effect on Sniffing [F(3,35)=4.645, P=0.041] and Total interaction time F(3,35)=4.712, P=0.037], there was no significant effect on Mounting F(3,35)=1.369, P=0.089] and Grooming partner [F(3,35)=1.134, P=0.108]. Subsequent Turkey analysis revealed that NET/PreCTL decreased Sniffing and Total interaction time by 27% and 27%, respectively, vs. VEH/PreCTL group, p<0.05. RSV completely reversed this effect, and there was no effect on Mounting (p=0.078) and Grooming partner (p=0.114).
